# Studies of aqueous U(iv) equilibrium and nanoparticle formation kinetics using spectrophotometric reaction modeling analysis†

**DOI:** 10.1039/d0ra05352j

**Published:** 2020-10-06

**Authors:** Wansik Cha, Hee-Kyung Kim, Hyejin Cho, Hye-Ryun Cho, Euo Chang Jung, Seung Yeop Lee

**Affiliations:** Nuclear Chemistry Research Laboratory, Korea Atomic Energy Research Institute 989-111 Daedeok-daero, Yuseong-gu Daejeon 34057 Republic of Korea wscha@kaeri.re.kr; Radioactive Waste Management Research Division, Korea Atomic Energy Research Institute 989-111 Daedeok-daero, Yuseong-gu Daejeon 34057 Republic of Korea

## Abstract

Hydrolysis of tetravalent uranium (U(iv)) and U(iv)-nanoparticle formation kinetics were examined over a wide range of temperatures using spectrophotometric reaction modeling analysis. The characteristic absorption bands representing U^4+^, U(OH)^3+^, and a proposed oxohydroxo species were newly identified in the UV region (190–300 nm). Dynamic absorption band changes in the UV and visible regions (360–800 nm) were explored to reevaluate the binary ion interaction coefficients for U(iv) ions and the thermodynamic constants of the primary hydrolysis reaction, including complexation constants, enthalpy, and entropy. No further hydrolysis equilibrium beyond the formation of U(OH)^3+^ was identified. Instead, an irreversible transformation of U(iv) ions to U(iv)-nanoparticles (NPs) was found to occur exclusively *via* the formation of a new intermediate species possessing characteristic absorption bands. The kinetic analysis, based on a two-step, pseudo-first-order reaction model, revealed that the rate of the initial step producing the intermediates is highly temperature-dependent with the measured kinetic energy barrier of ∼188 kJ mol^−1^. With additional experimental evidence, we conclude that the intermediates are oligomeric oxohydroxo U(iv) species occurring from the condensation of U(iv) ions and simultaneously participating in the nucleation and growth process of UO_2_(cr)-NPs.

## Abstract 2

The primary hydrolysis equilibrium of U^4+^ and the kinetics of U(iv)-nanoparticle formation were investigated by using spectrophotometric reaction modeling analysis and the spectral data collected in the UV and visible regions.

## Graphical abstract

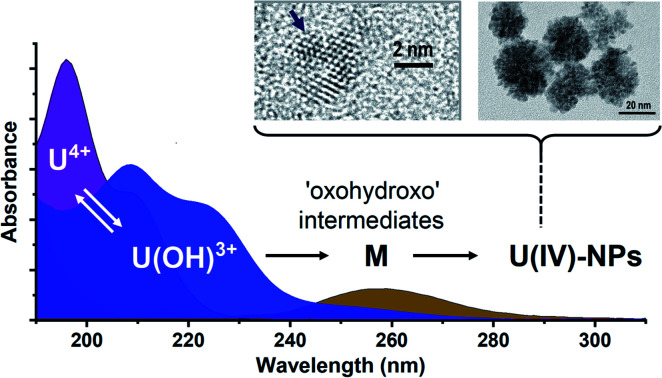

## Introduction

Understanding the physicochemical behaviors of radionuclides, such as the transport, solubility, and speciation in geomedia, is a prerequisite for the reliable safety assessment of nuclear disposal systems.^1,2^ Tetravalent uranium (U(iv)) is the main component of oxide nuclear fuels and the major reduced form of uranium in the natural environment, particularly under anoxic and reducing conditions of deep groundwater systems. However, understanding U(iv) speciation in solution is still a challenging task. Though efforts have been made to compile thermodynamic data of U(iv), there are relatively few speciation studies reported for aqueous phase reactions.^3–7^ Easy atmospheric oxidation of soluble U(iv) species and low solubility of U(iv) hydroxides and oxides have imposed experimental difficulties. In addition, the strong Lewis acid property of U(iv) renders U(iv) ions susceptible to being hydrolyzed and to form condensed species through olation and oxolation, depending on pH and temperature.^8–10^ These processes, as well as other complexations in a given system, often occur simultaneously to release protons. Thus, individual equilibria cannot be readily distinguished by solely monitoring pH changes (*e.g.*, potentiometry) in reaction media. This complication, referred to as “proton ambiguity,” has hampered understanding of the fundamental thermodynamic properties of reaction equilibria involving actinide (An) aqua ions and their hydrolysis products.^11^ Partly for this reason, among the stability constants of U(iv) hydrolysis the one for 1 : 1 complex (U(OH)^3+^) based on spectroscopic measurements is currently accepted in NEA-TDB.^3^

The condensation process can influence the apparent solubility of U(iv) to accelerate its distribution in the environment by producing insoluble uraninite (UO_2_(cr)) as well as oxo-hydroxo nanoclusters and inorganic colloids.^12,13^ Recent studies regarding the microbe-mediated redox transformation of U(vi) to U(iv) showed that nanometer- or submicrometer-sized crystalline particles (*i.e.*, uraninite) could be created *via* both biotic and abiotic pathways.^14–16^ More recently, Knope *et al.* identified a series of U(iv) nanoclusters of well-defined structures in the presence of carboxylic ligands by controlling reaction parameters like pH and temperature.^8^ We have also previously developed a hydrothermal U(iv)-nanoparticle (NP) preparation method directly from aqueous solutions, without organic ligands, to investigate their colloidal properties and interactions with organic molecules.^17^ It was found that U(iv)-NP formation is a temperature-sensitive process consuming monomeric U(iv) ions and producing pseudo-stable nanoclusters (approximately 20–30 nm) consisting of smaller crystalline-UO_2_ primary particles (2–6 nm). Inspired by this observation, we are currently interested in understanding the nature of the U(iv)-NP formation kinetics and its formation mechanism.

Spectrophotometry is a versatile detection technique for the speciation of An ions in a wide range of concentrations. The 5f^2^ electronic configuration of U^4+^ provides various energy levels that are detectable in the visible (Vis) region (*e.g.*, ϵ_(648 nm)_ ≈ 60 cm^−1^ M^−1^).^18^ It has been shown that, when coupled with a liquid-waveguide capillary cell (LWCC) setup, spectrophotometric analysis is possible for An ions down to μM-levels.^19^ To minimize the condensation of U(iv) toward polynuclear species, maintaining lower concentrations of uranium is advantageous. The absorption bands of U^4+^ and U(OH)^3+^ are distinguishable but often overlap each other. An early study by Cohen and Carnall reported the absorption spectra of U(iv) in DClO_4_ solution between 200 and 2600 nm.^20^ Later works for determination of the U(OH)^3+^ stability constant, including the one reported by Kraus and Nelson, used visible spectral bands (350–1100 nm) and have been reliably referred to date.^21,22^ The peaks in the visible region are attributed to the f–f electronic transitions from the ground state (^3^H_4_) to the upper states (^3^P_*n*_, ^1^I_6_, ^1^G_4_, ^1^D_2_, *n* = 0–2) arising from the f^2^ configuration.^23,24^ However, no previous work reported applicable absorption bands in the UV region for U(iv) speciation, while the transition to ^1^S_0_ at 245 nm has been explored by Kirishima *et al.* for the luminescence study of aqua U^4+^ ions.^25^

The objectives in the present study are threefold: (1) to employ the newly-found strong absorption bands in the UV region for speciation of U(iv) ions, (2) to determine and reevaluate the thermodynamic constants of the 1 : 1 hydrolysis reaction by using both absorption bands in UV and visible regions, and finally (3) to investigate NP formation kinetics and examine spectroscopic evidence for the presence of other U(iv) species, such as 1 : 2–1 : 4 hydrolyzed complexes or condensed polynuclear species. This approach has allowed us to identify new U(iv) species, potentially oxohydroxo intermediates, of which formation kinetics significantly depend on temperature. The thermodynamic constants regarding 1 : 1 hydrolysis and the binary ion interaction coefficient for U(iv) ions are also determined. Additionally, a simplified reaction model and the activation energy of the initial step of U(iv)-NP formation reaction are discussed.

## Experimental

### Sample preparation

A stock solution of U(vi) perchlorate was prepared from uranium dioxide, as described elsewhere.^26^ Next, U(iv) stock solutions (∼70 mM) were obtained by electrochemically reducing the acidified U(vi) stock solution (<pH 0.5) using a Hg working electrode.^20^ The U(vi) content in the U(iv) stock solution was monitored using both time-resolved laser fluorescence spectroscopy and inductively coupled plasma atomic emission spectroscopy (ICP-AES) analyses (<1% of the total U content).^27^ Sodium perchlorate (Sigma-Aldrich, 99%) was purified by recrystallization before use. All chemicals, including NaOH and HClO_4_, were of ultrapure reagent grade and used as received (Sigma-Aldrich and J.T. Baker for NaOH and HClO_4_, respectively). Electrolyte stock solutions were filtered through a 0.2 μm syringe filter (PVDF, Millipore) before use. All solution preparations, pH control, and sampling were carried out in an Ar-filled glove box to avoid CO_2_ dissolution unless otherwise noted.

Each sample subjected to spectrophotometric analysis was prepared fresh (daily) and batch-wise to have a fixed ionic strength while the solution pH varied within a series of samples. The ionic strength (*I*) of individual samples was controlled by adjusting the volume ratio of HClO_4_ and NaClO_4_ stock solutions. The addition of NaOH solution was done only into a U(iv) stock solution so that the final pH of each test sample was controlled either by diluting U(iv) stock solutions or by adding a standardized HClO_4_ solution.

A combination glass electrode (Orion Ross, Fisher Sci.) was used to determine the solution pH_c_ (−log[H^+^]), typically after the experiment. The electrode was pre-calibrated using standard HClO_4_ solutions of 0.01 and 0.0001 m (molal concentration) at the designated temperature of a given experiment. The electrode calibration curves yielded near-Nernstian slopes. When the solution pH_c_ was lower than 1.6, at which the electrode shows emf drifting and liquid junction potential, the [H^+^] of a sample solution was directly calculated with acidity and additive volumes of the U(iv) stock and HClO_4_ solutions used for sample preparation. Final concentrations of the U(iv) solutions for spectrophotometry were in the range of 0.1–2.5 mM. pH_c_ ranged from 0 to 2.2 and 2.0 to 3.0 for thermodynamic and kinetic analyses, respectively.

### UV-Vis spectrophotometry

Absorption spectra were measured using two types of spectrometer systems: a dual-beam UV-Vis spectrophotometer (Cary 100, Agilent), for both UV (190–340 nm) and visible (360–800 nm) regions, and a multi-channel spectrometer (MCS601, Carl Zeiss) for the visible region only. All visible spectra were baseline-corrected against a water blank. For the UV spectra, a series of blank solutions containing the same concentrations of HClO_4_ and NaClO_4_ as those of the corresponding samples were used for correction. Samples prepared in an Ar glove box were sealed into screw-capped quartz cells of different optical path-lengths (OPL): 1 mm and 10 mm for UV and Vis absorption measurements, respectively. In addition, an LWCC (WPI) with an OPL of 100 mm was used for visible spectra at room temperature (RT = 26 ± 1 °C). When measured using an LWCC setup, the sample solutions were introduced *via* a flow-through setup equipped with a syringe pump. Otherwise, the temperature of the quartz cells was precisely controlled in a cell holder (±0.05 °C, qChanger6, and Qpod 2e, Quantum Northwest).

### Monitoring U(iv)-NP formation kinetics

In this study, the conversion rate from U(iv) ions to U(iv)-NP was measured using sets of spectra collected over time. The relatively fast reaction kinetics at high temperatures were monitored using the multi-channel spectrometer capable of collecting spectra at various time intervals (down to ms-levels). For kinetic studies, an aliquot of U(iv) stock solution was filled into a gas-tight syringe (100 μL, Hamilton). It was then injected into a quartz cell filled with the appropriate electrolyte solution that was pre-equilibrated to the desired temperature (30–80 °C). In this way, the initiation of the reaction and the kinetic data collection began simultaneously.

After a hydrothermal reaction, *e.g.*, 5 h at 80 °C, the solution color changed from light blue to dark yellow. The synthesized U(iv)-NPs were characterized by high-resolution transmission electron microscopy (HR-TEM, JEOL, JEM 2001F) and dynamic light scattering (Zetasizer Nano ZS, Malvern Instruments) measurements for analysis of particle size, morphology, and crystalline structure.

### Spectrophotometric data analysis

To systemically interpret the multivariate spectrophotometric data, including experimental parameters like pH and concentrations, we employed a global analysis method, *i.e.*, a numerical data fitting procedure involving reaction modeling for both studies of aqueous equilibria and U(iv)-NP formation kinetics. For the analysis of hydrolysis equilibria, a series of absorption spectra of U(iv) as a function of pH_c_ were obtained from the samples prepared at a given condition, *i.e.*, ionic strength and temperature. Then, the conditional stability constant and singular spectra of the absorbing species involved in a given equilibrium model were derived using a commercial spectrophotometric data deconvolution program (HypSpec®2014, Protonic Software). The kinetic reaction analysis was performed similarly; by collecting a series of spectra over time and using a spectral data fitting program (ReactLab®™ Kinetics, JPlus Consulting Ltd.) to obtain the reaction rates and singular spectra of species involved in a given reaction model. For the kinetic data analysis, the molar absorption spectrum of U(OH)^3+^ was provided as a known parameter. Factor analysis and evaluation of the deconvolution were conducted beforehand to check the validity of the proposed reaction models using the function included in the softwares. Typically, the spectra of a full wavelength range, *i.e.*, 190–340 nm or 360–800 nm, were employed for both thermodynamic and kinetic data analyses. In this work it is assumed that the singular spectra of U(iv) species at a given temperature do not vary depending on the solution ionic strength. Three or more sets of sample series were examined to obtain the statistical results, including standard deviations.

### Calculation of thermodynamic constants

The thermodynamic constants of the first hydrolysis equilibrium (eqn (1)), including the stability constant log ^*^β_1_ and the changes in molar enthalpy (Δ_r_*H*_m_) and molar entropy (Δ_r_*S*_m_) of the reaction, were derived from the spectroscopic analysis as shown above.

1 U^4+^ + H_2_O(l) ⇄ U(OH)^3+^ + H^+^

The measured conditional stability constant given by2
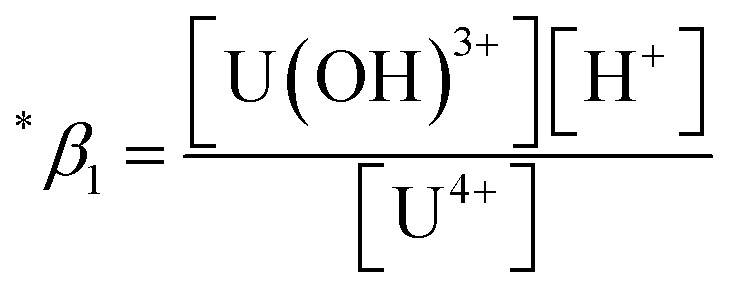
was converted to the standard constants 
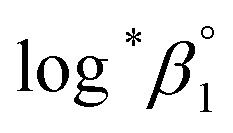
 at the standard condition (*I* = 0, *T* = 25 °C) using the specific ion interaction theory (SIT).^3^ Detailed conversion procedures are described in the ESI†. For the correction of *I*, molar concentrations (M) were converted to molal concentrations (m) using the conversion process described in NEA-TDB.^3^

In this work, we applied the following SIT formula for 1 : 1 U(iv) hydrolysis to obtain 
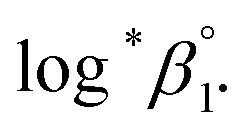
3
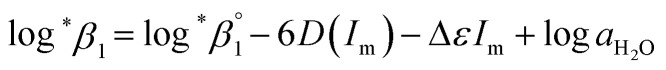
or3-1
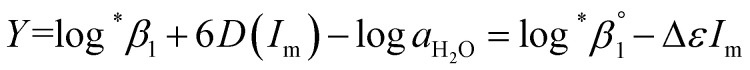


The values of log_10_ *a*_H2O_ and the calculation procedure of the Debye–Hückel term (*D*(*I*_m_)) are provided in the ESI (see also Tables S1 and S2†). The binary ion interaction coefficients (*ε* or Δ*ε*) used in this study are provided in Table 1. When a set of log ^*^β_1_ values exclusively depending on ionic strength were available, 
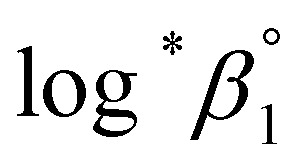
 alone or both 
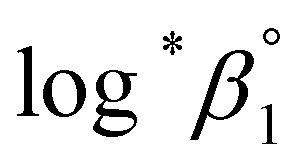
 and Δ*ε* were measured graphically by analyzing the intercept and slope of the *Y vs. I*_m_ plot (eqn (3-1)). The limiting values of log ^*^β_1_ at various temperatures are denoted collectively as 
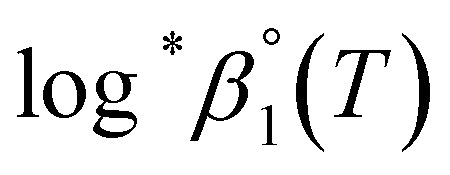
 (see ESI†). The uncertainty of the determined thermodynamic constants was expressed with the standard error at a 95% confidence level.

**Table 1:** Values of *ε*_ij_ used for SIT formula given by eqn (3)

*ε*_ij_ (kg mol^−1^) (j = ClO_4_^−^)	i = H^+^	i = U^4+^	i = U(OH)^3+^
	(0.14 ± 0.02)^a^	(0.76 ± 0.06)^a^	(0.48 ± 0.08)^a^
			(0.41 ± 0.07)^b^
Δ*ε* = *ε*(UOH^3+^, j) + *ε*(H^+^, j) − *ε*(U^4+^, j) = −(0.14 ± 0.05)^a^			
Δ*ε*′ = *ε*(UOH^3+^, j) − *ε*(U^4+^, j) = −(0.35 ± 0.03)^b^			

a Adopted from ref. 3.

b Determined in the present work.

## Results and discussion

### Monitoring absorption spectra of U(iv) ions

**Fig. 1:**
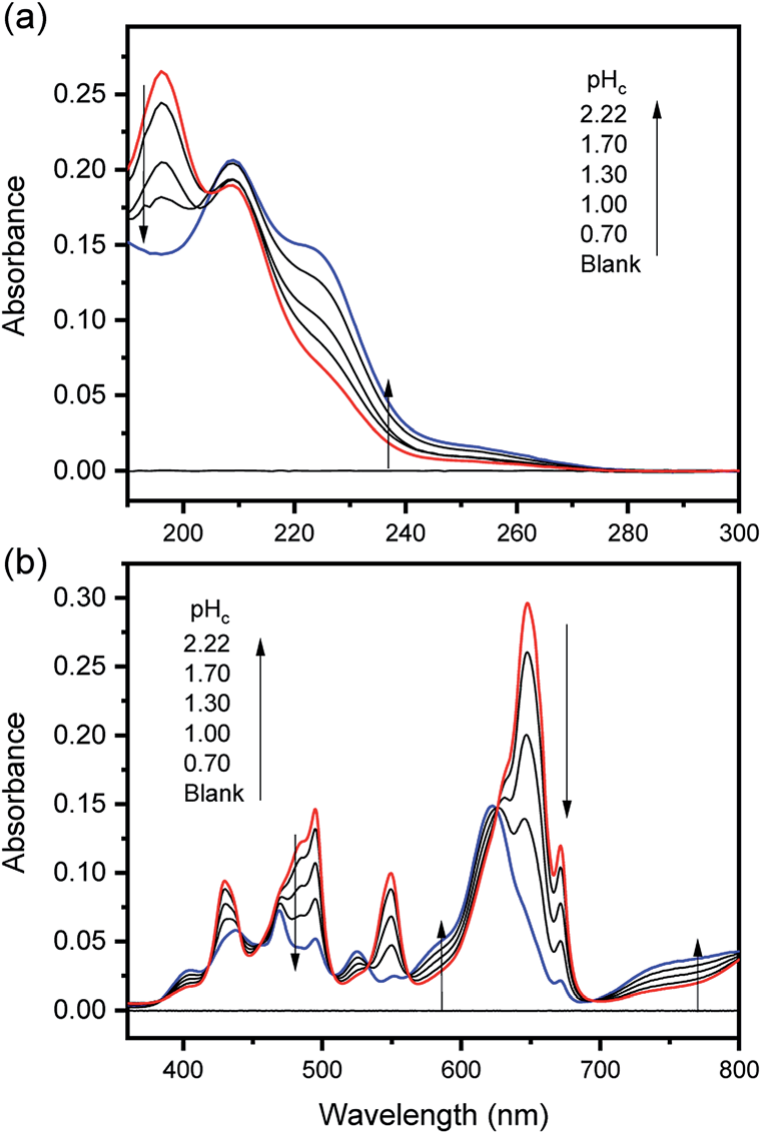
Changes in the absorption spectrum of aqueous solutions containing U(iv) (0.7 mM) at RT in the (a) UV and (b) visible regions (OPL is 1 mm and 100 mm, for UV and visible regions, respectively; *I* = 0.2 m).

**Fig. 2:**
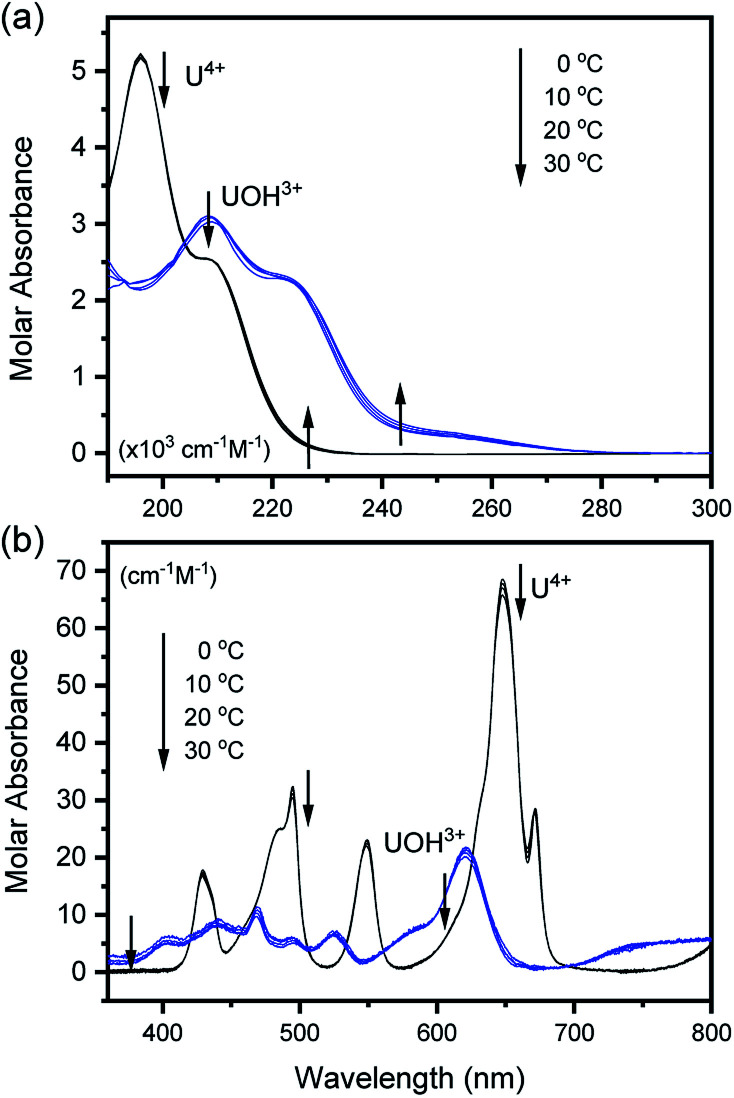
Deconvoluted molar absorption spectra of U^4+^ and U(OH)^3+^ in the (a) UV and (b) visible regions at various temperatures.

The characteristic absorption spectra of U^4+^ and U(OH)^3+^ were confirmed in both UV and visible regions, as shown in Fig. 1 and 2. The absorption peaks in the visible region are almost identical to those reported in the literature.^18,20,25^Fig. 1 shows pH-dependent spectral changes in both the UV and visible regions of sample solutions containing U(iv) (*I*, 0.2 m). At low pH_c_, U^4+^ ions are dominant, and the spectra show *λ*_max_ at 196 nm and 648 nm. As the solution pH_c_ increases, the absorption peaks of U(OH)^3+^ ions gradually emerge; the *λ*_max_ of the UV region red-shifts to 209 nm and that of the visible region blue-shifts to 621 nm. We emphasize that the versatile spectral changes in the UV region, depending on U(iv) speciation, is reported here for the first time. It should be noted that these spectral changes are reversible, according to the change of solution pH_c_ in both UV and visible regions, so that the forward and backward reactions of eqn (1) quickly achieve equilibrium.

To examine the thermodynamic properties of eqn (1), a set of spectra was collected from multiple samples (typically four to seven samples) under the same experimental parameters, except pH_c_, which typically varied from 0–2.2. The spectrum set was then analyzed using a numerical data fitting procedure (see Experimental section). To obtain the best fitting results of low uncertainty, we initially provided a measured spectrum of U^4+^ as a ‘known’ parameter, which was independently obtained in 2 M HClO_4_. Then, the molar absorption spectra of U^4+^ and U(OH)^3+^ were obtained from the converged outputs of the data fitting iteration process.

Fig. 2 summarizes those molar absorption spectra determined using the aforementioned process. As expected, the factor analysis result of the individual set of spectral data confirmed that no further U(iv) species, other than U^4+^ and U(OH)^3+^, is involved in the hydrolytic equilibrium under the given conditions in this work. However, since the reaction products irreversibly occurring at high pH or an elevated temperature can complicate the monitoring of the equilibria (see following sections), the sample solutions for the thermodynamic study were examined only at temperatures ≤ 30 °C. The *λ*_max_ and molar absorption coefficient (ϵ, *Y*-axis of Fig. 2) of the observed individual absorption peaks and isosbestic points are summarized in Table 2.

**Table 2:** Absorption maxima and molar absorptivities of U(iv) species measured in aqueous NaClO_4_ solutions at 25 °C^a^

Species	U^4+^		Isosbestic points		U(OH)^3+^		Intermediate
Region	*λ*_max_ (nm)	ϵ (cm^−1^ M^−1^)	*λ* (nm)	ϵ (cm^−1^ M^−1^)	*λ*_max_ (nm)	ϵ (cm^−1^ M^−1^)	*λ*_max_ (nm)
UV	196	5110 ± 90	204	2730 ± 120	209	3060 ± 60	—
	210^sh^	2500 ± 110			225^sh^	2200 ± 50	
					247^sh^	310 ± 40	260 ± 3
Vis	429	16.9 ± 0.7	419	4.5 ± 0.4	400^sh^	3.9 ± 0.1	
			440	7.2 ± 0.5	439	6.8 ± 0.1	429 ± 1
	485^sh^	24.9 ± 0.9	457	5.9 ± 0.3	468	9.4 ± 0.1	
	495	30.7 ± 0.8	506	3.6 ± 0.3	494	4.9 ± 0.1	468 ± 1
			533	3.9 ± 0.3	525	5.9 ± 0.1	
	549	22.2 ± 0.7	561	3.4 ± 0.2	583^sh^	7.0 ± 0.1	544 ± 1
	648	66.5 ± 1.8	625	19.0 ± 0.4	621	20.2 ± 0.3	625 ± 1
	671	28.1 ± 0.7	695	0.9 ± 0.1	739^sh^	4.4 ± 0.2	669 ± 1

a ^sh^Indicating a shoulder peak.

As shown in Fig. 1(b) and 2(b), the structure of the visible absorption of the U^4+^ aqua ion has the characteristic of an electronic transition within the 5f^2^ configuration (f → f). The characteristic six bands in the visible region are attributed to transitions from the ground state (^3^H_4_) to the upper states (^3^P_*n*_, ^1^I_6_, ^1^G_4_, ^1^D_2_, *n* = 0–2, see Fig. S1†). The ϵ of these bands is relatively small (<70 cm^−1^ M^−1^) because the f–f transition is a Laporte (or parity change)-forbidden transition. In contrast, the broad bands appearing in the UV region are intense (2–5 × 10^3^ cm^−1^ M^−1^) since both the 5f^2^ → 5f^1^6d^1^ transition and charge-transfer (CT) process, known to be responsible for absorption in the UV region, are Laporte-allowed.^18,28,29^

Interestingly, Hashem *et al.* recently reported the spectra in the UV region for UCl_5_^−^ and UBr_5_^−^ measured in tetrahydrofuran (THF).^30^ While more red-shifted, *i.e.*, *λ*_max_ is at 280–320 nm, those spectra share a similar spectral shape with that of U(OH)^3+^ (*λ*_max_ at 209 nm). The possible contribution of the transition to the lowered 5f^1^6d^1^ energy level and/or the halide-to-uranium CT process was pointed out in the study. In addition, Miles demonstrated the relationship between the wavelength of the CT band edge of aqueous An ions and their redox potentials early on.^31^ It is considered that H_2_O or OH^−^ can participate in a CT process as a charge acceptor or donor, respectively. Similarly, we speculate that the electronic transitions responsible for the spectra of U^4+^ and U(OH)^3+^ in the UV region (Fig. 2(a)) possess characteristics of the transition from ^3^H_4_ (5f^2^) to 5f^1^6d^1^ and/or the CT process between H_2_O/OH^−^ and U(iv). It is further confirmed that mere changing of the isotope (H/D) in the aqueous solutions, *i.e.*, using D_2_O, has negligible effects on the band positions of the absorption spectra (Fig. S2†). Rather, replacing the aqueous media with organic media such as alcohols induces solvatochromism, *i.e.*, spectral red-shift (data not shown), which probably arises from differences in the dipolarity, polarizability, and hydrogen bond donating (or lone pair donating) capacity of the solvent molecules. These features can be considered typical of a CT process, however, a more in-depth study is needed to elucidate the nature of the U(iv) ion absorption bands in the UV region.

On the other hand, it is noteworthy that the sharp peak at 245 nm, which was assigned to the ^3^H_4_ → ^1^S_0_ transition in the literature,^25^ is absent in Fig. 1(a) and 2(a). This phenomenon is probably due to the relatively low ϵ values at 245 nm so that the peak could not be observed in the scale of molar absorbance in Fig. 1(a) and 2(a). In addition, as shown in Fig. 2, there is almost no temperature dependency in the molar absorption spectra, except for a slight decrease in ϵ of the major absorption bands when the temperature increases. Nonetheless, we used the individual molar absorption spectrum, deconvoluted at each temperature, for further analysis of temperature-dependent spectral data.

### Evaluation of thermodynamic constants

As described in Experimental section, each value of log ^*^β_1_ derived from the global fitting procedure was converted to 
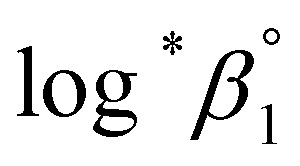
 or 
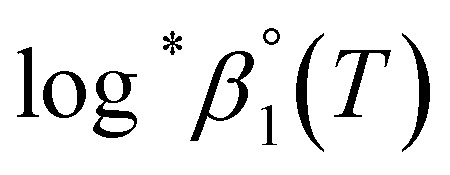
 based on the SIT formula, as shown in eqn (3). In all experiments, we acquired two types of log ^*^β_1_ in parallel from each data set in the UV and visible regions. As summarized in Test A of Table 3, two 
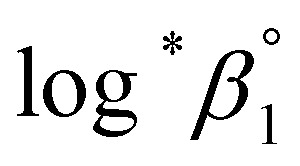
 values from UV and visible spectra agreed well with each other within their uncertainty. Thus, the results from UV and visible spectra are combined to produce statistically averaged values for the rest of the thermodynamic study. The average value of 
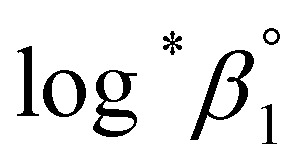
 determined is −(0.51 ± 0.05), which is located at the upper boundary of the reported value: −(0.54 ± 0.06).^3^ Nonetheless, there is a slight but systematic difference between the results from the UV and visible spectra; 
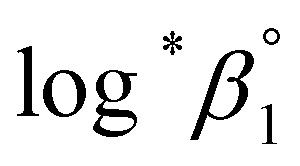
 from the UV data is approximately 0.02 higher than those from visible-region data (see Table 3). While the reason is not yet clear, we think that the background correction process applied for UV data may increase the contribution of U(OH)^3+^ in the final spectrophotometric data.

**Table 3:** Summary of the thermodynamic constants obtained for 1 : 1 hydrolysis of the aqua U^4+^ ion

Test	pH_c_	*I* (mol kg^−1^)	*T*, °C	log ^*^β_1_	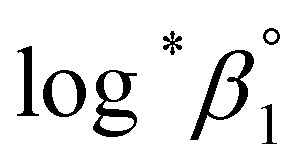	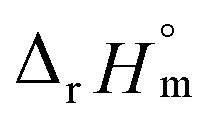 (kJ mol^−1^)	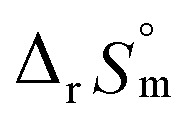 (J K^−1^ mol^−1^)
A	1.7–2.2	0.02	RT	−(0.94 ± 0.04)	−(0.51 ± 0.05)	—	—
	1.3–2.2	0.05		−(1.05 ± 0.05)			
	1.0–2.2	0.10		−(1.16 ± 0.04)			
	0.7–2.2	0.20		−(1.24 ± 0.03)	(−(0.50 ± 0.06)^UV^, −((0.52 ± 0.08)^Vis^)^a^		
	0.3–2.2	0.51		−(1.38 ± 0.04)			
	0–2.2	1.05		−(1.48 ± 0.03)			
	0.8–2.1	2.09		−(1.56 ± 0.02)			
B	0.82–2.10	0.15	0	−(1.87 ± 0.03)	−(1.17 ± 0.03)^b^	43.4 ± 3.0	136 ± 11
			5	−(1.72 ± 0.03)	−(1.02 ± 0.03)^b^		
			10	−(1.58 ± 0.03)	−(0.87 ± 0.03)^b^	(41.6 ± 2.8)^c^	(117 ± 10)^c^
			15	−(1.46 ± 0.05)	−(0.75 ± 0.05)^b^		
			20	−(1.32 ± 0.03)	−(0.61 ± 0.03)^b^		
			25	−(1.20 ± 0.02)	−(0.48 ± 0.03)^b^		
			30	−(1.07 ± 0.02)	−(0.34 ± 0.03)^b^		
C	1.70	0.02–1.05	0–30	(See Fig. 4(b))	(See Fig. 4(c))	(See Table S4 and Fig. S4)	
Ref. 3		0.02–2.21	25		−(*0.54 ± 0.06)	46.9 ± 9.0	147 ± 30

a Each 
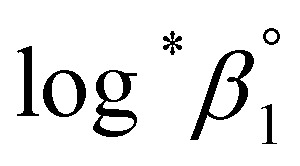
 was derived exclusively from one of the spectral data sets (UV or visible region).

b Values are 
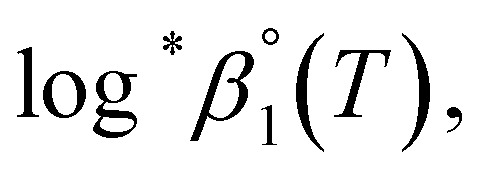
 which were obtained at each temperature (see Experimental section).

c Conditional constants at *I* = 0.15 m, *i.e.*, Δ_r_*H*_m_ and Δ_r_*S*_m_, as obtained using log^*^β_1_.

**Fig. 3:**
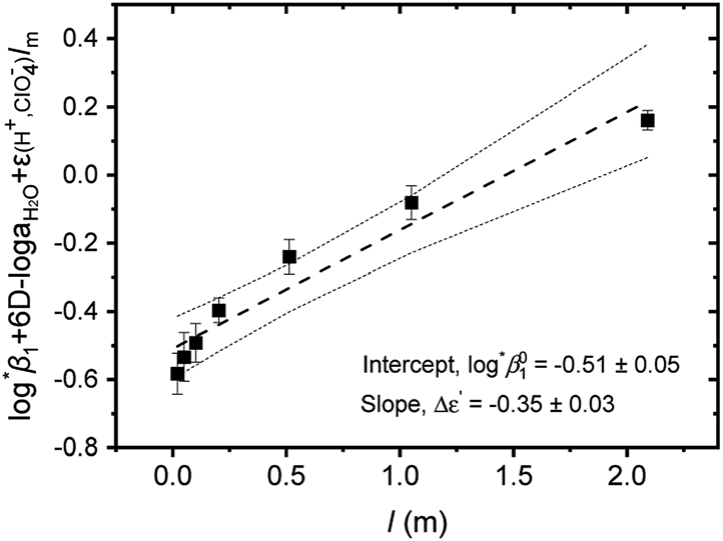
Extrapolation to *I* = 0 of log ^*^β_1_ data of Test A in Table 3 for the hydrolysis of U^4+^ to U(OH)^3+^ using the SIT formula given by eqn (4). (■) experimental data, (---) linear fit, and (⋯) 95% confidence bands.

Further, we attempted to reevaluate the binary interaction coefficients (*ε*) of U(iv) ions by using the sets of log ^*^β_1_ in Table 3. As listed in Table 1, the uncertainty of *ε*(H^+^, ClO_4_^−^) is relatively small, *i.e.*, ±0.02.^3^ Therefore, to yield the best result in terms of uncertainty we focused on obtaining Δ*ε*′, the direct difference between each *ε* of U^4+^ and U(OH)^3+^, using4
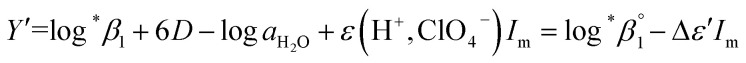
a modified form of eqn (3-1). Fig. 3 displays the *Y*′ *vs. I*_m_ plot from eqn (4). By applying non-weighted linear regression fitting analysis, we obtain 
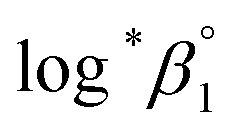
 = −(0.51 ± 0.05) from the *y*-intercept and Δ*ε*′ = −(0.35 ± 0.03) from the slope. If we adopt the *ε*(U^4+^, ClO_4_^−^) = 0.76 ± 0.06 from NEA-TDB, the new *ε*(U(OH)^3+^, ClO_4_^−^) is estimated as 0.41 ± 0.07, as summarized in Table 1, which is within in the range of previously reported values.^3^ Also, in Table S3† the reestimated Δ*ε* is also compared with those from the literature.^3,4^

In addition, the changes of reaction enthalpy and entropy were examined by monitoring log ^*^β_1_ at various temperatures. As shown in Fig. S3,† the spectra of U(iv) aqueous solution varies significantly upon temperature changes indicating the temperature dependency of log ^*^β_1_. These spectral changes remain reversible at temperatures ≤ ∼30 °C and pH < ∼2, depending on the electrolyte level. For Test B in Table 3, a set of samples having an identical *I*_m_ (0.15 m) were prepared, but the pH_c_ of individual samples varied in a range of 0.82–2.10. Thus, log ^*^β_1_ was calculated at each temperature as implemented in Test A. Subsequently, log ^*^β_1_ was converted to 
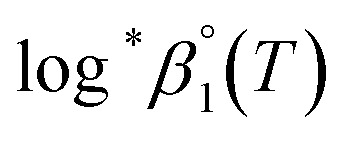
 at each temperature by applying the newly determined Δ*ε*′ in Table 1 and the SIT formula (eqn (3)).

**Fig. 4:**
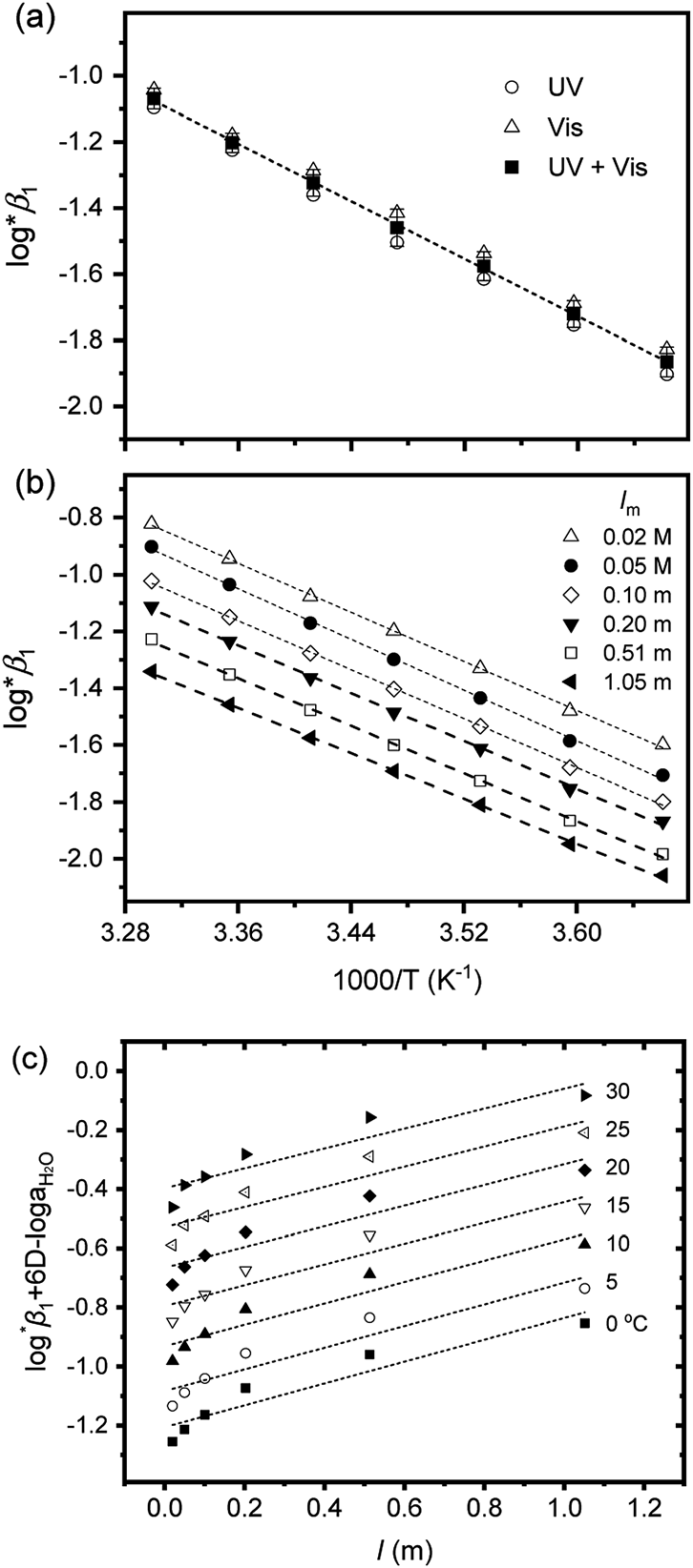
Two van't Hoff plots using log ^*^β_1_ in Table 3, (a) from Test B at *I* = 0.15 m and (b) from Test C at pH_c_ 1.70 (ln *K* = ln 10 × log ^*^β_1_ in eqn (5)). (c) Extrapolation to *I* = 0 of log ^*^β_1_ data of (b) data using the SIT formula, eqn (3-1), at each temperature. All dotted lines indicate linear fit results.

On the other hand, for Test C, a set of samples having the same pH_c_, but differing in *I*_m_ in a range of 0.02–1.05 m, were examined. In this case, log ^*^β_1_ was directly extracted from the individual spectra; [U^4+^] and [U(OH)^3+^] were directly determined by finding the best combinational fitting of the experimental spectrum using the molar absorption spectra in Fig. 2 and an Excel Solver function-based optimization process developed in this study. The temperature dependences of log ^*^β_1_ from Tests B and C in Table 3 are demonstrated in Fig. 4(a) and (b), respectively, using the van't Hoff equation,5
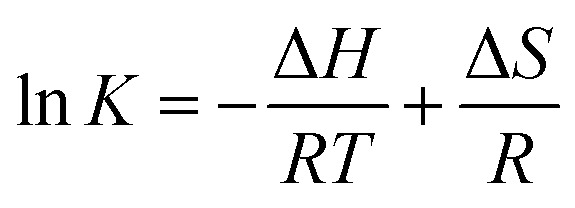
where *R* is the ideal gas constant (8.31446 J K^−1^ mol^−1^), *T* is the temperature in kelvin (K), and *K* is the conditional equilibrium constant measured at each temperature (*i.e.*, ln *K* = ln 10 × log ^*^β_1_).

For Test B, two conditional constants, *i.e.*, Δ_r_*H*_m_ and Δ_r_*S*_m_ at *I* = 0.15 m, can be determined from the graphical analysis of van't Hoff plots in Fig. 4(a), which correspond to 41.6 ± 2.8 kJ mol^−1^ and 117 ± 10 J K^−1^ mol^−1^, respectively. Further, by employing 
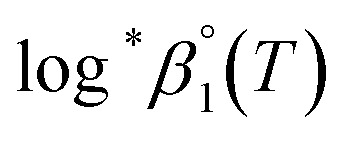
 the standard molar thermodynamic constants, 
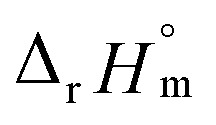
 and 
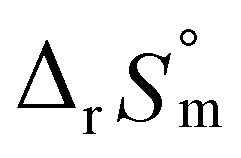
 were determined to be 43.4 ± 3.0 kJ mol^−1^ and 136 ± 11 J K^−1^ mol^−1^, respectively, which are all in good agreement with literature values within the uncertainty range.^3^ Although Fig. 4(a) exhibits a systematic difference in the determined constants depending on the wavelength region (UV or Vis) of spectral data, the differences 
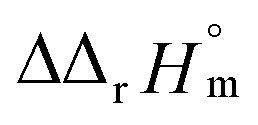
 and 
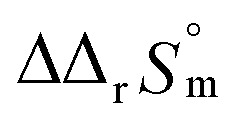
 are within the range of measured uncertainty: approximately 1.3 kJ mol^−1^ and 3 J K^−1^ mol^−1^, respectively. The negative slopes of van't Hoff plots in Fig. 4 clearly indicate the endothermic property of the 1 : 1 hydrolysis reaction. The large positive value of 
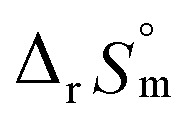
 should result from the reduction of total charges upon hydrolysis (+4 → +3) of the U(iv) ion. The less restrained water molecules or the liberation of some from U(iv) ion's hydration shell contribute to increase in the disorder of the system.^22^

Test C and its corresponding results, as shown in Fig. 4(b), demonstrate the particular importance of the ionic strength correction for the determination of 
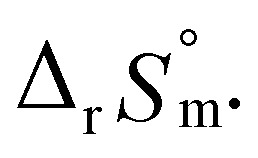
. It is apparent in Fig. 4(b) that the slope of the plot, *i.e.*, Δ_r_*H*_m_, does not fluctuate significantly. However, the intercept, Δ_r_*S*_m_, consistently decreases as *I*_m_ increases (see also Table S4†). Therefore, by applying the ionic strength correction to the data in Fig. 4(b), the 
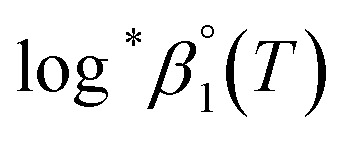
 at each temperature was obtained, as displayed in Fig. 4(c). A new van't Hoff plot 
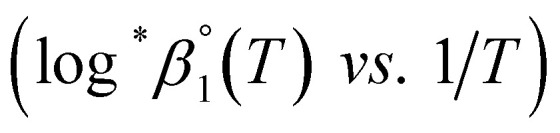
 was then used to calculate 
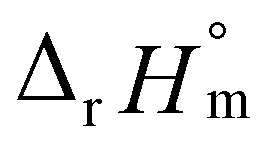
 and 
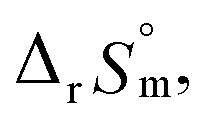
, as shown in Fig. S4.† After the ionic strength correction, the determined values for 
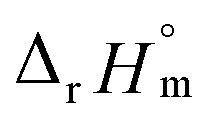
 and 
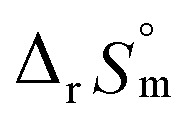
 fall well within the uncertainty range of the values from Test B, which are the representative 
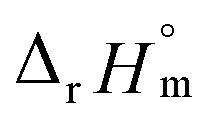
 and 
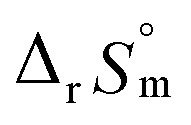
 of this work. Also, these measured constants are compared in Table S5† with the selected literature values. Interestingly, Fig. 4(c) also allowed us to estimate the temperature dependency of Δ*ε* from the slope analysis of the plots. In fact, Δ*ε* at each temperature is similar to each other, and their standard deviation is approximately 0.01. Thus, we conclude that the Δ*ε* change over the temperature range (0–30 °C) in this work plays a minor role in the thermodynamics of the 1 : 1 hydrolysis reaction.

### Reevaluated thermodynamic constants using literature data

We further attempted to reevaluate the thermodynamic constants that were recently reported by Brown and Ekberg using the selected literature data.^4^ All the available stability constants at 25 °C in the literature and those in Table 3 were combined and plotted as a function of the ionic strength (see Fig. S5†). The Δ*ε* and 
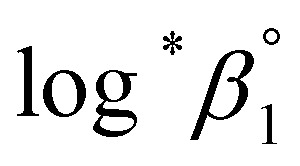
 were derived by analyzing the non-linear *I*-dependence of log^*^β_1_ using a two-parameter SIT model (Δ*ε* = Δ*ε*_1_ + Δ*ε*_2_ log *I*).^4^ For the reaction enthalpy and entropy, the stability constants at *I* = 0 in Table 3 and those selected from the literature (Fig. S6†) were combined, so that the overall temperature varied over a wide range (0–150 °C). The reevaluated constants are listed in Table 4.

**Table 4:** Reevaluated thermodynamic constants using literature values for 1 : 1 hydrolysis of the aqua U^4+^ ion

Reference	Δ*ε*_1_, Δ*ε*_2_^a^ (kg mol^−1^)	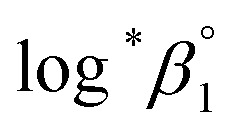 a	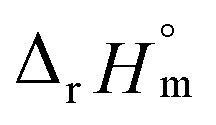 b (kJ mol^−1^)	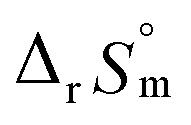 b (J K^−1^ mol^−1^)
Ref. 4	−(0.28 ± 0.03), 0.28 ± 0.06	−(0.58 ± 0.08)	42.7 ± 3.3	135 ± 10
This work^c^	−(0.30 ± 0.05), 0.32 ± 0.08	−(0.59 ± 0.08)	43.3 ± 2.2	136 ± 7

a Based on the two-term SIT expression in ref. 4; Δ*ε* = Δ*ε*_1_ + Δ*ε*_2_ log *I*.

b Used values of 
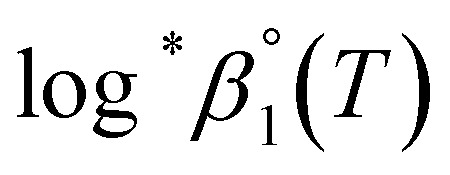
 at 0 ≤ *T* ≤ 150 °C.

c See calculation details in Fig. S5 and S6.

### Kinetic analysis at elevated temperatures

**Fig. 5:**
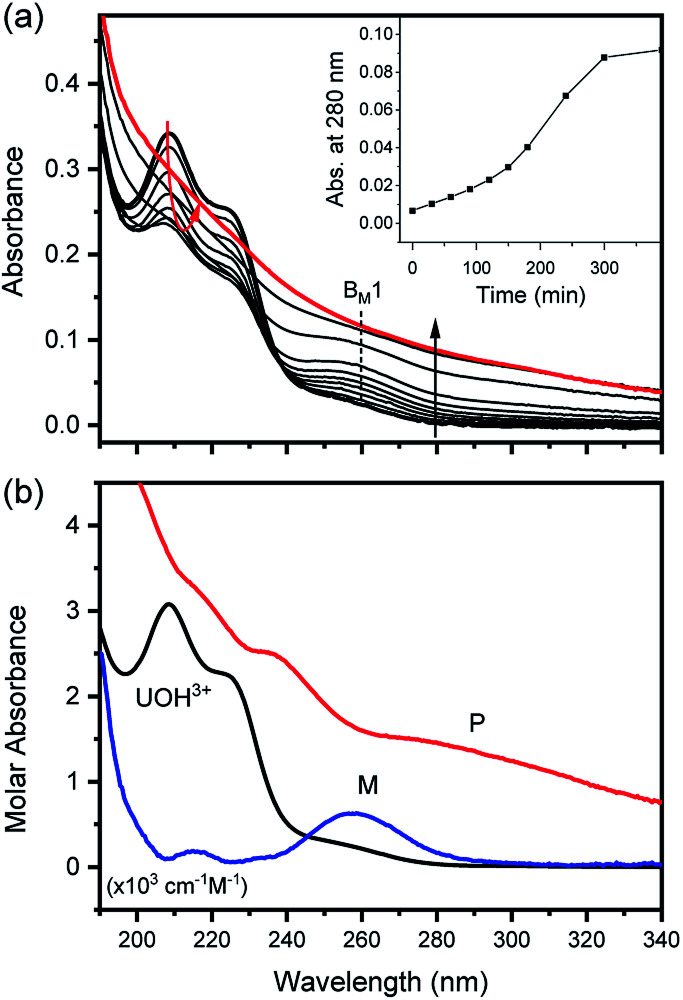
Spectrophotometric kinetic analysis of the U(iv)-NP formation reaction at 26 °C and pH_c_ 3.0 (*I* = 0.1 m, [U(iv)] = 0.12 mM, and OPL = 1 mm). (a) Experimental spectra collected in the UV range (inset shows absorbance change at 280 nm) and (b) singular spectra of U(OH)^3+^, M (intermediates), and P (U(iv)-NPs) obtained from reaction kinetic analysis. Note that M and P spectra are not to scale, and B_M_1 indicates one of the major absorption bands of M.

**Fig. 6:**
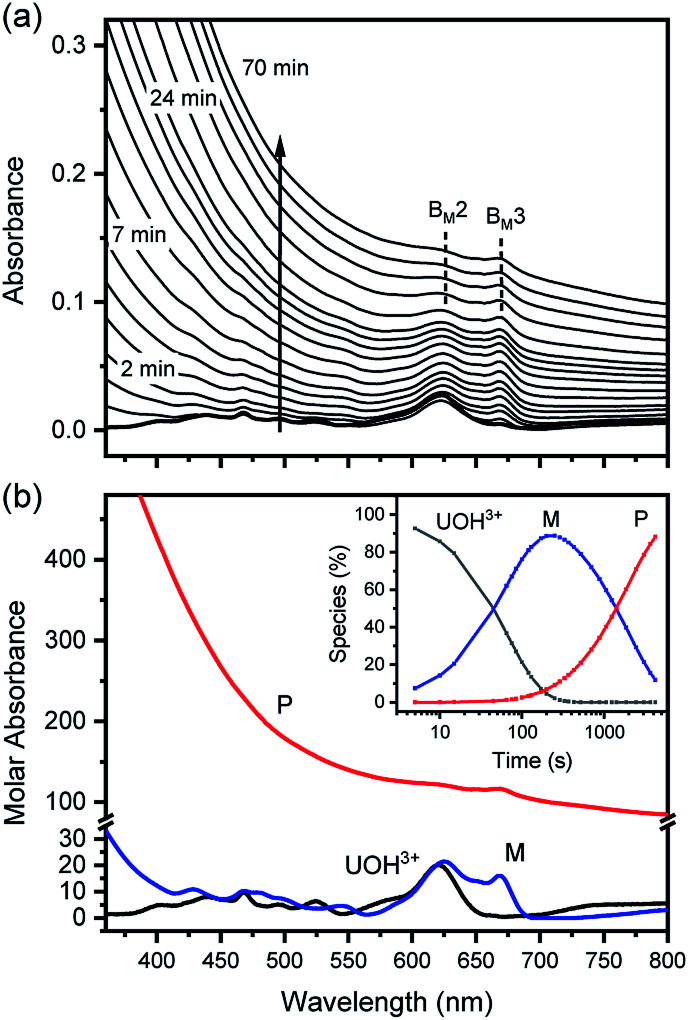
Spectrophotometric kinetic analysis of U(iv)-NP formation reaction at 70 °C and pH_c_ 2.0 (*I* = 0.1 m, [U(iv)] = 1.2 mM, and OPL = 10 mm). (a) Experimental spectra collected in the visible range and (b) singular spectra of U(OH)^3+^, M, and P obtained from reaction kinetic modeling analysis; inset shows the optimized reaction profile. Note that M and P spectra are not to scale, and B_M_2 and B_M_3 are among the major absorption bands of M.

One aim of this work is to investigate the hydrolytic reaction of U(iv) at higher pH or temperature. Under such conditions, equilibria involving U(OH)_*n*_^+4−*n*^ (*n* ≥ 2) species are expected to be established according to their stability constants; for U(OH)_2_^2+^
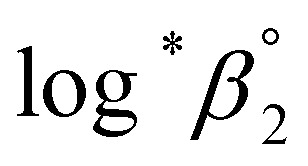
 = −(1.1 ± 1) and for U(OH)_3_^+^
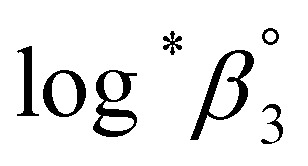
 = −(4.7 ± 1).^3^ As an extension of the 1 : 1 hydrolysis equilibrium shown above, we assume that further mononuclear ligations of hydroxide also result in facile and reversible reactions. If so, as soon as an aliquot of the U(iv) stock solution is added into an electrolyte with higher pH or temperature (at *t* = 0), as shown in Fig. 5 and 6, U(OH)^3+^ might instantaneously hydrolyze further into U(OH)_2_^2+^ or other forms to establish a new equilibrium. Fig. 5(a) and 6(a) display the results of spectrophotometric reaction monitoring over time in two different settings; Fig. 5(a) at 26 °C and a pH_c_ of 3 and Fig. 6(a) at 70 °C and a pH_c_ of 2. However, we could not find spectroscopic evidence of the occurrence of such higher hydrolyzed species within our observation window (typically from minutes to days). Despite the difference in the reaction parameters of the two experiments in Fig. 5(a) and 6(a), each initial spectrum is almost identical with that of U(OH)^3+^ in terms of the spectrum shape and molar absorbance.

In fact, only an irreversible and slow process of which the final product is U(iv)-NPs from the reactant U(OH)^3+^ was observed as the reaction proceeded. As shown in Fig. 5(a) and 6(a), the overall spectrum changes exhibit the characteristics of a kinetic process in which reaction rates depend on pH and temperature. The reaction shown in Fig. 6(a) is an example of the hydrothermal U(iv)-NP production process. As we reported previously, the synthesized U(iv)-NP can retain its colloidal stability up to several weeks at pH ∼ 2 and RT.^17^ Interestingly, the emergence of an intermediate species is discovered in this work during the conversion of aqua U(iv) ions to U(iv)-NPs. The intermediate (M) was first probed due to its characteristic bands in both UV and visible regions, denoted as B_M_1, B_M_2, and B_M_3 in Fig. 5(a) and 6(a). Since it is obvious that the conversion processes to U(iv)-NPs are far from equilibria, we established the simple reaction model6
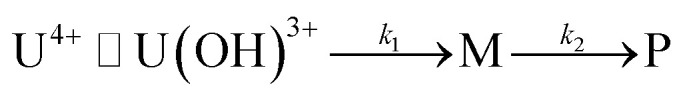
(M = oxohydroxo intermediates, P = U(iv)-NPs) to implement reaction kinetic analysis.

Herein we assume that the two stepwise reactions, U(OH)^3+^ to M and M to P, are pseudo first-order reactions. It is apparent that, as shown in Fig. 5(b) and 6(b), U(OH)^3+^ is the predominant species at *t* = 0 and plays a role as the reactant initiating the process. Therefore, *k*_1_ indicates the rate of consumption of U(OH)^3+^ in the system to possess quantitatively meaningful information. In contrast, since the chemical identity of M and P cannot be defined exactly, there is a limitation that the other outcomes of kinetic analysis will provide only qualitative information. In addition, light-scattering by the nanoparticles probably results in the deviation from the Beer–Lambert law for spectrophotometric analysis. However, despite such limitations, the optimization of kinetic analysis on the two different data sets was conducted successfully. It resulted in reliable converged outputs, *e.g.*, reaction rates and singular spectra of M and P, as shown in Fig. 5(b) and 6(b) (see also Fig. S7,† an example of the residual plot). The inset plot in Fig. 6(b), one of the results of kinetic analysis, clearly shows the evolution and consumption of M over time.

The singular spectra of M clearly exhibit the characteristic major bands at B_M_1, B_M_2, and B_M_3 (∼260, 625, and 669 nm, respectively) in addition to some minor bands (see Table 2). In fact, B_M_3 plays an important role in this work as it reveals the irreversible nature of the first step (*k*_1_). In a separate experiment, we quenched the reaction when [M] reached its maximum level (see the inset of Fig. 6(b)) by lowering the temperature or by adding HClO_4_ to see if a backward reaction occurs. In any case, M remained in the solution once formed (see Fig. S8†), *e.g.*, up to days at a refrigerated temperature (data not shown). Therefore, we confirm that the backward reaction of M to U(OH)^3+^ is not facile. On the other hand, the singular spectra of P are characterized by the monotonously decreasing absorption pattern from the UV to the visible region.

**Fig. 7:**
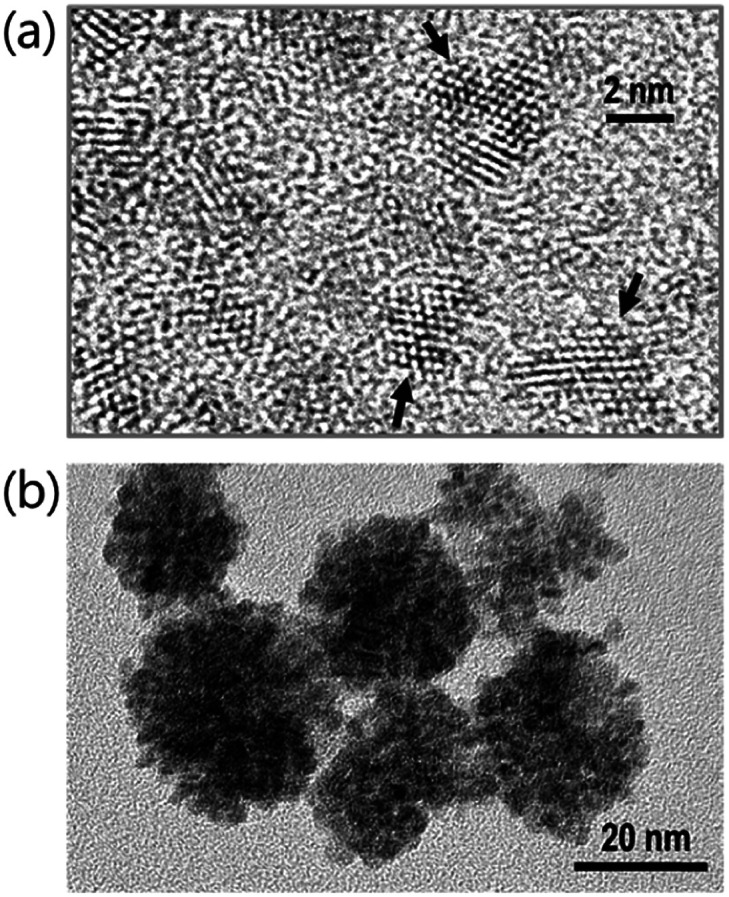
TEM images of (a) primary particles from the sample solution taken at the maximum level of [M] (see the inset of Fig. 6(b)) and (b) U(iv)-NPs (P), *i.e.*, the clusters of primary particles after the reaction (eqn (6)) is completed. Arrows indicate distinctive lattice fringes (*d* = 0.32 nm) of the nanoscale U(iv) crystallites.

Fig. 7(a) and (b) show TEM images, the direct evidences of crystalline primary particles (2–3 nm) of U(iv)-NPs and their clusters (20–30 nm), respectively. We confirmed that both particles possess uraninite-like crystalline structures. The Rayleigh scattering of the incident light by the growing U(iv)-NPs can account for the increasing absorption over time in a wide wavelength range. It is also noted that a few broad undulating features in UV-Vis absorption of P (Fig. 5(b) and 6(b)) can be attributed to the semiconductor properties of U(iv)-NPs, *e.g.*, UO_2_(cr) possessing optical bandgaps. Previous studies reported that such broad band absorption of UO_2_ could change by varying the U/O ratio and the oxidation state of U.^32,33^ Thus, we think that both of the primary particles and the U(iv)-NP clusters are to be responsible for the overall spectral shape of P. Therefore, P in the reaction model (eqn (6)) is used to represent the primary particles and their clusters combined.

The results of reaction rate analysis also show temperature dependency. Typically, the transformation of aqua U(iv) ions to U(iv)-NPs at RT is a slow process, as shown in the inset of Fig. 5(a), that can take a few hours to days, depending on the *I* and pH. The *k*_1_ and *k*_2_ obtained at RT are comparable with each other, ∼1–2 × 10^−4^ s^−1^. In contrast, at higher temperatures, as shown in Fig. 6, the overall reaction proceeds much faster and generally *k*_1_ > *k*_2_; *k*_1_ = (1.2 ± 0.3) × 10^−2^ s^−1^ and *k*_2_ = (5 ± 3) × 10^−4^ s^−1^ at 70 °C (pH = 2.0). Thus, in this situation, the last step (*k*_2_) of eqn (6) becomes the rate-determining step of the whole process. While both *k*_1_ and *k*_2_ can be influenced by the solution parameters, like pH, *I*, and temperature, we found that *k*_1_ is more susceptible to temperature changes.

**Fig. 8:**
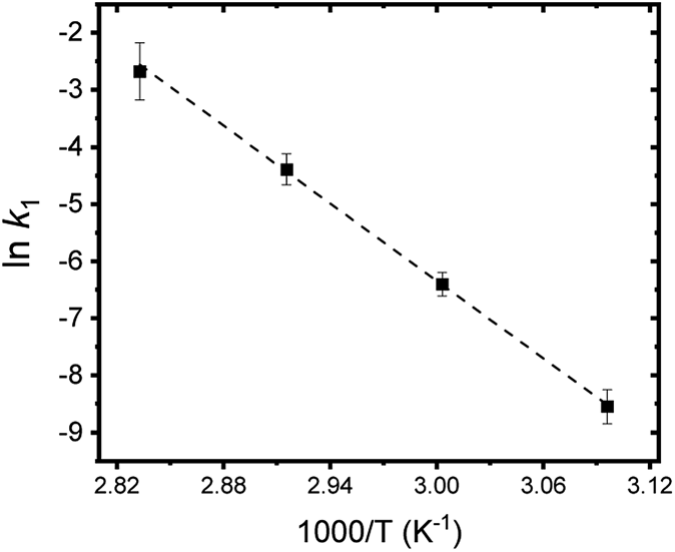
Arrhenius plot of ln *k*_1_*vs.* 1/*T*. A dotted line indicates the linear fit result.

Fig. 8 summarizes the results of monitoring *k*_1_ at different temperatures (50–80 °C) as a plot of ln *k*_1_*vs.* 1/*T*, an Arrhenius-type plot. Since *k*_1_ reflects the consumption rate of soluble U(iv) ions in eqn (6), it can be used to describe the nucleation rate (*J*) of particles as shown by7
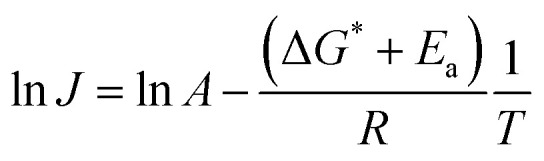
where *A* is the pre-exponential kinetic factor related to ion diffusion, and nuclei surface properties, and ΔG^*^ and *E*_a_ are the thermodynamic and kinetic energy barriers (J mol^−1^), respectively.^34,35^ ΔG^*^ is related to interfacial energies, and *E*_a_ is the apparent activation energy. Eqn (7) is based on the theoretical pathway where nuclei are formed by the addition of one monomer at a time until the nucleus grows large enough to stabilize the nucleus as a new phase.^35^ Here, ΔG^*^ is related to the interfacial energy of the new solid phase, and *E*_a_ is the apparent activation energy of the kinetic process. In this study, by employing a simple approach of assuming that *J* = *k*_1_ (J mol^−1^) and using the slope analysis of Fig. 8, the nucleation energy barrier (ΔG^*^ + *E*_a_) is determined to be ∼188 ± 25 kJ mol^−1^. The energy barrier is comparatively high, which is often observed in the crystalline metal-oxide growth processes^36–38^ (the individual values of ΔG^*^ and *E*_a_ have not been determined since it is beyond the scope of this work).

**Scheme 1:**
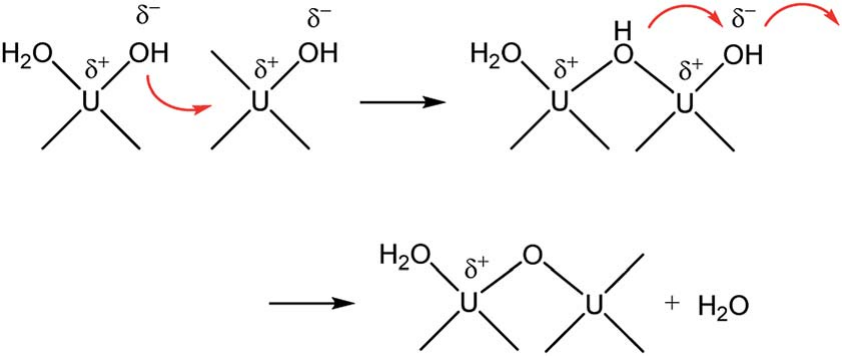
Condensation of U(iv) ions through a two-step associative mechanism as an initiating step of U(iv)-NP nucleation.

Therefore, combining all the evidences above, it is reasonable to conclude that M represents oligomeric oxohydroxo U(iv) species, *e.g.*, U_*x*_(O)_*y*_(OH)_*z*_^4*x*−2*y*−*z*^. Recently, Falaise *et al.* identified a hexameric U(iv) species (U_6_O_4_(OH)_4_^12+^) in aqueous solutions containing an amino acid under a condition that is similar to that of our study.^39^ They also reported that the hexamer possesses a traceable absorptivity (*λ*_max_ at 664 nm) at longer wavelengths than those of U^4+^ in the visible range. In fact, the overall process is very similar to that of iron oxide NP formation from soluble iron ions following a two-step oxolation mechanism.^40^ We speculate that the kinetic process represented by *k*_1_ and *E*_a_ in this study also includes a similar condensation process, as shown in Scheme 1. In this association mechanism, the lability of (coordinated) water molecules should be a key factor determining the reaction rate. Thus, it is very probable that the formation process of M is associated with both the condensation of U(iv) ions and the subsequent nucleation process that eventually creates primary particles.

In addition, it should be pointed out that in the M-dominant solution, the individual primary particles (crystallites) can exist separately from each other (Fig. 7(a)). However, at the last stage of the reaction (eqn (6)), raspberry-shape U(iv)-NP clusters become prevalent, as shown in Fig. 7(b). As U(OH)^3+^ and M are depleted in solution, the second step of Scheme 1 seems to prevail and contribute to reducing surface charges *via* oxo-bridge formation, thereby increasing the surface energy of primary particles and finally induce their aggregation. Therefore, we speculate that M is likely the key species involved in the nucleation and growth of these primary particles *via* influence on their colloidal stability.

## Conclusions

To summarize, we examined the U(iv) hydrolytic reaction at a wide range of temperatures using UV-Vis spectrophotometric analysis. Characteristic absorption bands newly identified in the UV region (190–300 nm) were employed for U(iv) speciation together with those in the visible region. Aided by a numerical global fitting analysis for the collected spectral data, we successfully reevaluated the thermodynamic constants of the 1 : 1 hydrolysis reaction and the ion interaction coefficient for U(iv) ions with ClO_4_^−^. The new values reported in this work include 
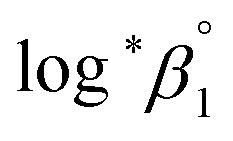
 = −(0.51 ± 0.05), 
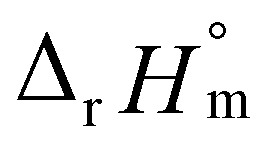
 = 43.4 ± 3.0 kJ mol^−1^, and 
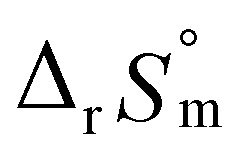
 = 136 ± 11 J K^−1^ mol^−1^, which are well matched with literature values within the uncertainty range. Further, kinetic studies demonstrate that the increase of pH and/or temperature primarily induces the irreversible formation of reaction intermediates and subsequent production of U(iv)-NPs. The reaction intermediates that are newly observed in this study are suggested to be oligomeric oxohydroxo U(iv) species. This interpretation is strongly supported by other results, such as the confirmed two-step reaction model, the electronic absorption characteristics of the isolated spectrum of the intermediate, and the high energy barrier of the intermediate species generating step. We believe that the formation process of the intermediate is comprised of both the condensation of U(iv) ions and the subsequent nucleation process of U(iv)-NPs, while the interpretation of kinetic data should be considered only in a qualitative manner since the chemical identity of the intermediate and nanoparticles cannot be defined exactly. We expect that the spectroscopic and kinetic properties of various U(iv) species provided in this work will play a crucial role in understanding their speciation and evolution into polymeric oxohydroxo species in aqueous environments.

## Conflicts of interest

There are no conflicts to declare.

## Supplementary Material

RA-010-D0RA05352J-s001

## References

[cit1] Altmaier M., Gaona X., Fanghänel T. (2013). Chem. Rev..

[cit2] Maher K., Bargar J. R., Brown G. E. (2013). Inorg. Chem..

[cit3] Guillaumont R. , Fanghänel T., Fuger J., Grenthe I., Neck V., Palmer D. A. and Rand M. H., Update on the chemical thermodynamics of uranium, neptunium, plutonium, americium and technetium, in (OECD, NEA-TDB): Chemical Thermodynamics, Elsevier, North-Holland, Amsterdam, 2003, vol. 5, pp. 187, 707–752

[cit4] Brown P. L. and Ekberg C., Actinide Metals, in Hydrolysis of Metal Ions, Wiley-VCH, Germany, Weinheim, 2016, ch. 9, vol. 1, pp. 336–349

[cit5] Grenthe I. , Wanner H. and Forest I., Chemical thermodynamics of uranium, in (OECD, NEA-TDB): Chemical Thermodynamics, Elsevier, North-Holland, Amsterdam, 1992, vol. 1, pp. 120–124

[cit6] Rai D., Felmy A. R., Ryan J. L. (1990). Inorg. Chem..

[cit7] Neck V., Kim J. I. (2001). Radiochim. Acta.

[cit8] Knope K. E., Soderholm L. (2013). Chem. Rev..

[cit9] Natrajan L. S., Swinburne A. N., Andrews M. B., Randall S., Heath S. L. (2014). Coord. Chem. Rev..

[cit10] Zänker H., Hennig C. (2014). J. Contam. Hydrol..

[cit11] Hummel W. , Anderegg G., Rao L., Puigdomenech I. and Tochiyama O., Chemical Thermodynamics of Compounds and Complexes of U, Np, Pu, Am, Tc, Se, Ni and Zr with Selected Organic Ligands, in (OECD, NEA-TDB): Chemical Thermodynamics, Elsevier, North-Holland, Amsterdam, 2005, vol. 9

[cit12] Walther C., Denecke M. A. (2013). Chem. Rev..

[cit13] Kim J.-I. (2006). Nucl. Eng. Technol..

[cit14] Lee S. Y., Cha W., Kim J.-G., Baik M. H., Jung E. C., Jeong J. T., Kim K., Chung S. Y., Lee Y. J. (2014). Chem. Geol..

[cit15] Bargar J. R., Bernier-Latmani R., Giammar D. E., Tebo B. M. (2008). Elements.

[cit16] Jeon B.-H., Dempsey B. A., Burgos W. D., Barnett M. O., Roden E. E. (2005). Environ. Sci. Technol..

[cit17] Cho H., Cha W. (2019). Colloids Interfaces.

[cit18] Carnall W. T. in Gmelin Handbook of Inorganic Chemistry, ed. K.-C. Buschbeck, Springer-Verlag Berlin Heidelberg GmbH, 1989, pp. 69–161

[cit19] Richard E. W., Yung-Jin H., Heino N. (2005). Radiochim. Acta.

[cit20] Cohen D., Carnall W. T. (1960). J. Phys. Chem..

[cit21] Kraus K. A., Nelson F. (1950). J. Am. Chem. Soc..

[cit22] Kraus K. A., Nelson F. (1955). J. Am. Chem. Soc..

[cit23] Kirishima A., Kimura T., Tochiyama O., Yoshida Z. (2003). Chem. Commun..

[cit24] Crosswhite W. T. C. M. , in The Chemistry of the Actinide Elements, ed. J. J. Katz, G. T. Seaborg and L. R. Morss, Springer, Dordrech, 1984, pp. 1235–1277

[cit25] Kirishima A., Kimura T., Nagaishi R., Tochiyama O. (2004). Radiochim. Acta.

[cit26] Jung E. C., Cho H.-R., Park K., Yeon J.-W., Song K. (2009). Appl. Phys. B.

[cit27] Cha W., Lee S., Jung E., Cho H. R., Baik M. (2014). J. Radioanal. Nucl. Chem..

[cit28] Ryan J. L., Jørgensen C. K. (1964). Mol. Phys..

[cit29] Jørgensen C. K. , in Progress in Inorganic Chemistry, ed. S. J. Lippard, New York, 1970, pp. 101–152

[cit30] Hashem E., Swinburne A. N., Schulzke C., Evans R. C., Platts J. A., Kerridge A., Natrajan L. S., Baker R. J. (2013). RSC Adv..

[cit31] Miles J. H. (1965). J. Inorg. Nucl. Chem..

[cit32] Ackermann R. J., Thorn R. J., Winslow G. H. (1959). J. Opt. Soc. Am..

[cit33] Jones J. M., Murchison D. G. (1965). Nature.

[cit34] Li Q., Jun Y.-S. (2018). Commun. Chem..

[cit35] Lasaga A. C. , Kinetic Theory in the Earth Sciences, Princeton University Press, Princeton, New Jersey, 1998

[cit36] Bretos I., Jiménez R., Ricote J., Calzada M. L. (2018). Chem. Soc. Rev..

[cit37] Deb P., Basumallick A. (2004). J. Nanopart. Res..

[cit38] Zhang H., Banfield J. F. (2002). Chem. Mater..

[cit39] Falaise C., Neal H. A., Nyman M. (2017). Inorg. Chem..

[cit40] Thanh N. T. K., Maclean N., Mahiddine S. (2014). Chem. Rev..

