# Supplementary material for: Studies of aqueous U(iv) equilibrium and nanoparticle formation kinetics using spectrophotometric reaction modeling analysis
Source: RSC Adv. 2020 Oct 6;10(60):36723–33. doi: 10.1039/d0ra05352j (PMC9057037; doi:10.1039/d0ra05352j)
Supplement: RA-010-D0RA05352J-s001 [file RA-010-D0RA05352J-s001.pdf]

Electronic Supplementary Material (ESI) for RSC Advances.
This journal is © The Royal Society of Chemistry 2020

Supporting Information

Studies of Aqueous U(IV) Equilibrium and Nanoparticle Formation Kinetics Using Spectrophotometric Reaction Modeling Analysis

Wansik Cha[*a], Hee-Kyung Kim[a], Hyejin Cho[a], Hye-Ryun Cho[a], Euo Chang Jung[a], Seung Yeop Lee[b]

[*]Corresponding author (e-mail: wscha@kaeri.re.kr)

[*a]Nuclear Chemistry Research Laboratory, [b]Radioactive Waste Management Research Division, Korea Atomic Energy Research Institute, 989-111 Daedeok-daero, Yuseong-gu, Daejeon 34057, Republic of Korea

[Ionic strength correction using SIT formula]

The measured conditional stability constant, $\log{}^*\beta_1$ was converted to the standard constants, $\log{}^*\beta_1°$ at the standard condition (I = 0, T = 25°C) by correcting the effects of I and adopting activity coefficient terms ($\log\gamma$) as shown in Eq. (S1).

$$^*\beta_1 = \frac{[\mathrm{U(OH)}^{3+}][\mathrm{H}^+]}{[\mathrm{U}^{4+}]}$$

$$\log{}^*\beta_1 = \log{}^*\beta_1^{o} + \log\gamma_{U^{4+}} - \log\gamma_{UOH^{3+}} - \log\gamma_{H^+} + \log a_{H_2O} \qquad \text{(S1)}$$

Equilibria involving H_2O(l) as a reactant or product require a correction for the water activity (a_{H2O}). The $\log_{10} a_{\mathrm{H2O}}$ were calculated from the a_{H2O} values at 25 °C provided in NEA-TDB for $NaClO_4$ (see Table S1). The SIT formula for activity coefficients is:

$$\log_{10}\gamma_i = -z_i^2 D(I_m) + \sum_j \varepsilon(i,j)m_j \qquad \text{(S2)}$$

where z_i is the charge of ion i.

$$D(I_m) = \frac{A\sqrt{I_m}}{1 + Ba_d\sqrt{I_m}} \qquad \text{(S3)}$$

is a Debye-Hückel term where I_m is the molal ionic strength; A and B are Debye-Hückel constants; a_d is the size or effective diameter of the ion in angstrom; $B \cdot a_d$ in Eq. (S3) is considered as a constant (= 1.5). In Eq. (S2), the binary ion interaction coefficients (ε) for the pair of species i and j are assumed to be equal to zero for neutral species or for ions of the same sign. Thus, for the cations involved in 1:1 U(IV) hydrolysis the summation in Eq. (S1) is restricted to the ClO_4^- counter ion (= j) that was chosen for ionic strength control in this work. The values of a_{H2O} and A, $B \cdot a_d$ used in this work are listed in Tables S1 and S2, which were extracted from the literature. For the $B \cdot a_d$ and a_{H2O} terms we assume that these values are constant within the temperature range (0–30 °C) in this study, thus the values at RT in Tables S1 and S2 were used for the calculation of $\log{}^*\beta_1°$ at other temperatures, i.e., $\log{}^*\beta_1°$(T).

[Tables]

Table S1. Water activity used in this study (see Eq. (S1)).

I (m)	a_{H_2O}
0.02	0.9993
0.05	0.9983
0.10	0.9966
0.15	0.9948
0.51	0.9833
1.05	0.9660
2.09	0.9337

Note. Values for $NaClO_4$ media are adapted from NEA-TDB (Ref. [3] in the main text). Some values are extrapolated.

Table S2. Debye-Hückel constants used in Eq. (S3).

T (°C)	A	Ba_d
0	0.4913	
5	0.4943	
10	0.4976	
15	0.5012	1.5
20	0.5050	
25	0.5091	
30	0.5135	

Note. All values are adapted from Ref.[3] in the main text.

Table S3. Comparison of the binary ion interaction coefficient ($\Delta\varepsilon$) obtained in this work and those from literatures.

$\Delta\varepsilon^{a}$ (kg/mol)	References
-(0.14 ± 0.05)	Ref. [3]
-(0.21 ± 0.04)	this work[b]
$\Delta\varepsilon_1$ = -(0.28 ± 0.03)<br>$\Delta\varepsilon_2$ = 0.28 ± 0.06	Ref. [4]
$\Delta\varepsilon_1$ = -(0.30 ± 0.05)<br>$\Delta\varepsilon_2$ = 0.32 ± 0.08	this work[c]

[a] $\Delta\varepsilon$ at $I = 0$ (= $\varepsilon(UOH^{3+}, j) + \varepsilon(H^+, j) - \varepsilon(U^{4+}, j), j = ClO_4^-$).

[b] $\Delta\varepsilon$ was calculated by adding $\varepsilon(H^+, ClO_4^-)$ to $\Delta\varepsilon'$ presented in Table 1 in the main text.

[c] Based on the two-parameter SIT model in Ref. [4]; $\Delta\varepsilon = \Delta\varepsilon_1 + \Delta\varepsilon_2 \log I$

Table S4. Dependence of $\Delta_r H_m$ and $\Delta_r S_m$ on I. Each conditional constant was calculated from the individual van't Hoff plots ($\log {}^*\beta_1$ vs. 1/T) in Fig. 4(b).

Test (pH$_c$)	I (mol/kg)	T (°C)	$\Delta_r H_m$ (kJmol^{-1})	$\Delta_r S_m$ (JK^{-1}mol^{-1})
C (1.70)	0.02	0 ~ 30	41.4 ± 0.5	121 ± 2
	0.05		42.8 ± 0.6	124 ± 2
	0.10		41.3 ± 0.5	117 ± 2
	0.20		40.2 ± 0.6	111 ± 2
	0.51		40.1 ± 0.5	109 ± 2
	1.05		38.1 ± 0.4	100 ± 2

Table S5. Comparison of the reaction enthalpy and entropy data measured in this work and those from literatures for the first hydrolysis of U^{4+} in aqueous H/$NaClO_4$ solution.

T (°C)	I	$\Delta_r H_m$ (kJmol^{-1})	$\Delta_r S_m$ (JK^{-1}mol^{-1})	References
0 – 30	0	43.4 ± 3.0[a]	136 ± 11[a]	this work
	0.15 m	41.6 ± 2.8[a]	117 ± 10[a]	
10 – 150	0	46.9 ± 9.0	147 ± 30	Ref. [3]
25 – 150	0	42.9 ± 3.3[b]	135 ± 10[b]	Ref. [4]
0 – 150	0	43.3 ± 2.2[c]	136 ± 7[c]	this work
15.2 – 24.7	0.19 M	44.3 ± 6	126 ± 20	Ref. [S1], re-estimated in Ref.[5]
10, 43	0.5 M	46.9 ± 3	129 ± 10	Ref. [S2], re-estimated in Ref.[5]
25 – 100	0.5 m	42 ± 7	-	Ref. [S3], re-estimated in Ref.[5]
50 – 125	0.99 m	45 ± 7	-	
25 – 150	0	46 ± 4	143 ± 14	

[a] Data taken from Table 3 in the main text.
[b] Data calculated from the plot of Fig. S6 using $\log{}^{*}\beta_1^{\circ}$ values listed in Table 9.6 of Ref. [4].
[c] Reestimated values from the combined data from Table 3 and Ref. [4](see Fig. S6).

Table S6. Stability constants for the first hydrolysis of U^{4+}, which were used in Fig. S5.

Temp. (°C)	I (mol kg^{-1})	$\log^{*}\beta$ (accepted)	$\log^{*}\beta$ +6D-loga(H_2O)	Reference	
25	0.017	-(0.92 ± 0.17)	-0.587		
25	0.033	-(1.07 ± 0.17)	-0.634		
25	0.035	-(1.00 ± 0.17)	-0.553		
25	0.045	-(1.10 ± 0.17)	-0.608		
25	0.063	-(1.14 ± 0.17)	-0.582		
25	0.111	-(1.22 ± 0.10)	-0.540		
25	0.111	-(1.28 ± 0.10)	-0.600		
25	0.121	-(1.23 ± 0.10)	-0.530	Ref. [S4]	
25	0.121	-(1.29 ± 0.10)	-0.590		
25	0.274	-(1.35 ± 0.10)	-0.450		
25	0.513	-(1.53 ± 0.10)	-0.468		
25	0.534	-(1.50 ± 0.10)	-0.427		
25	0.534	-(1.49 ± 0.10)	-0.417		
25	0.565	-(1.44 ± 0.10)	-0.353		
25	1.06	-(1.54 ± 0.10)	-0.289		
25	2.21	-(1.59 ± 0.10)	-0.153		Ref. [4]
25	3.5	-(1.88 ± 0.20)	-0.331	Ref. [S5]	
25	2.21	-(1.64 ± 0.10)	-0.203	Ref. [S6]	
25	3.5	-(1.58 ± 0.10)	-0.031	Ref. [S7]	
24.7	0.192	-(1.08 ± 0.10)	-0.270	Ref. [S1]	
25	1.05	-(1.61 ± 0.09)	-0.361	Ref. [S8]	
25	3.5	-(1.55 ± 0.10)	-0.001	Ref. [S9]	
25	0.25	-(1.44 ± 0.20)	-0.564		
25	0.36	-(1.30 ± 0.20)	-0.330		
25	0.48	-(1.39 ± 0.20)	-0.345		
25	0.51	-(1.21 ± 0.20)	-0.149		
25	0.65	-(1.31 ± 0.20)	-0.186	Ref. [S3]	
25	0.75	-(1.44 ± 0.20)	-0.278		
25	0.86	-(1.54 ± 0.20)	-0.343		
25	0.99	-(1.68 ± 0.20)	-0.446		
25	1.09	-(1.72 ± 0.20)	-0.461		
26	0.02	-(0.94 ± 0.06)	-0.586		
26	0.05	-(1.05 ± 0.07)	-0.541		
26	0.101	-(1.17 ± 0.06)	-0.506	This work	
26	0.202	-(1.25 ± 0.04)	-0.425		
26	0.513	-(1.37 ± 0.05)	-0.311	(Table 3 of the main text)	
26	1.051	-(1.48 ± 0.05)	-0.228		
26	2.091	-(1.55 ± 0.03)	-0.131		

[Figures]

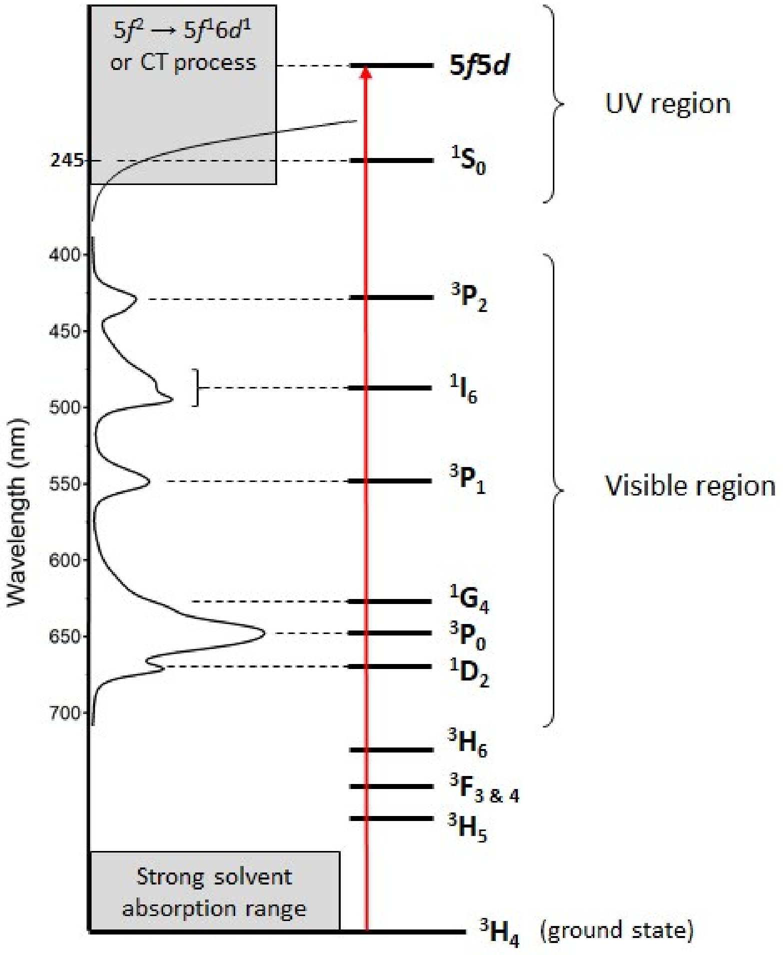

Fig. S1. Electronic energy states of U^{4+} ($5f^2$). The arrow denotes for the transition to the UV region. (adapted from Ref. [23] in the main text)

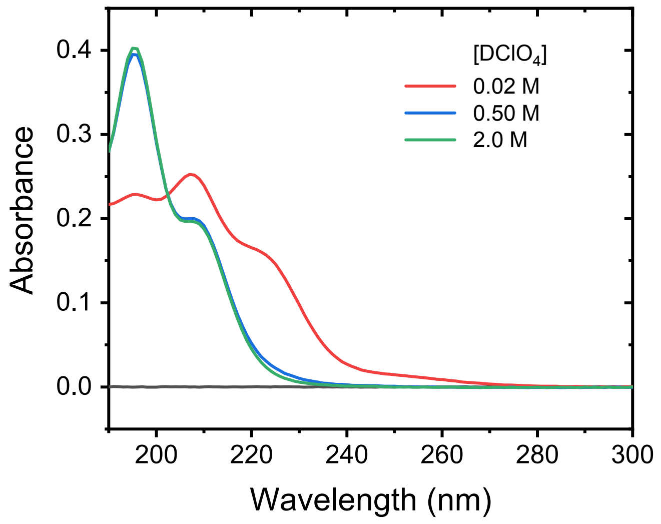

Fig. S2. Absorption spectra of D_2O solutions containing U(IV) (0.7 mM) in the UV region (OPL = 1 mm, I ~ [$DClO_4$], H/D in solution = ~ 0.01). The absorption spectra are nearly identical with those obtained in the $HClO_4/H_2O$ solution as shown in Figs. 1 and 2 in the main text.

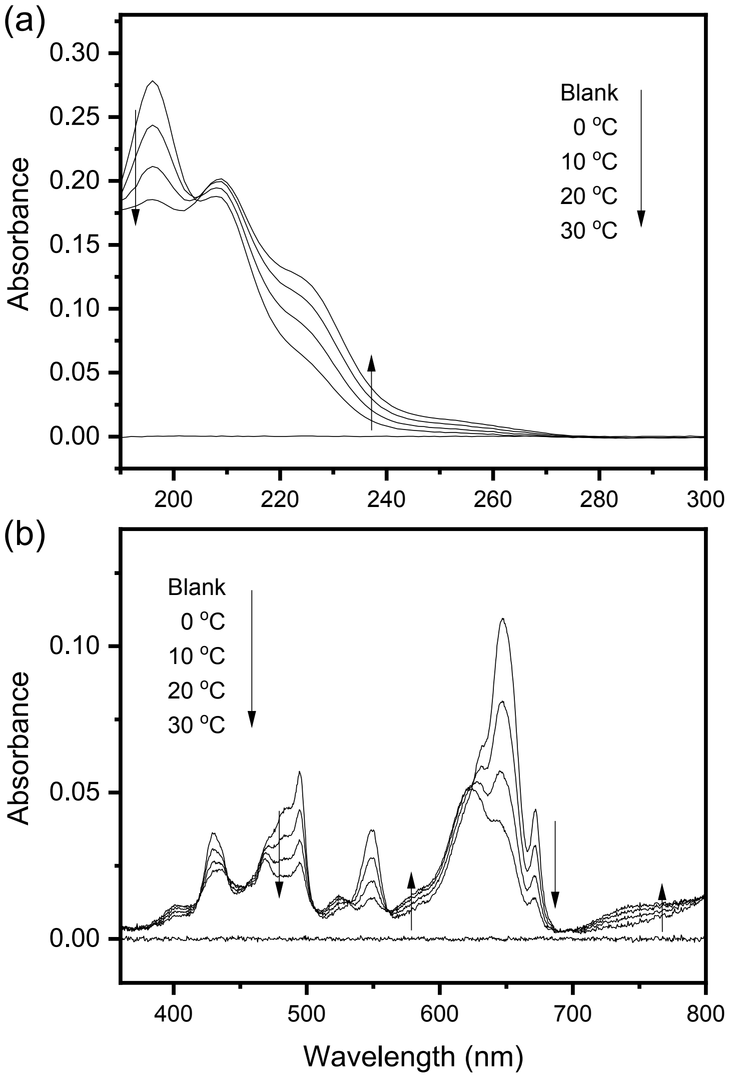

Fig. S3. Temperature-dependent changes of the absorption spectrum of aqueous solutions at $pH_c = 1.70$ and $I = 0.15$ m in the (a) UV region and (b) visible regions (OPL = 1 mm and 10 mm; [U(IV)], 0.7 mM and 2.8 mM, respectively).

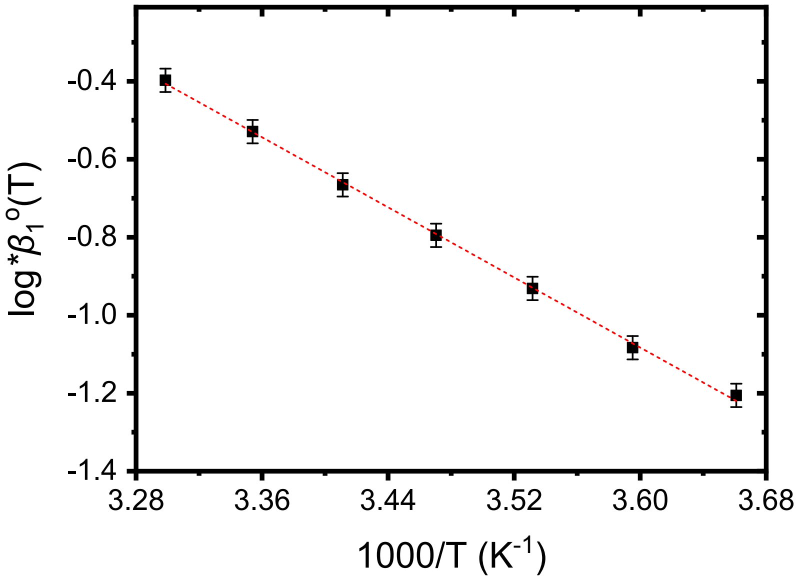

Fig. S4. Van't Hoff plot using $\log{}^*\beta_1^\circ(T)$ calculated from the ionic strength correction shown in Fig. 4(c) (Test C at pH_c = 1.70, 0 – 30 °C). Dotted lines indicate linear fit results. The standard state constants, $\Delta_r H_m^\circ$ and $\Delta_r S_m^\circ$, were determined to 43.0 ± 3.2 $kJmol^{-1}$ and 134 ± 11 $JK^{-1}mol^{-1}$, respectively, which are within the uncertainty range of the representative values in this work as shown in Table 3.

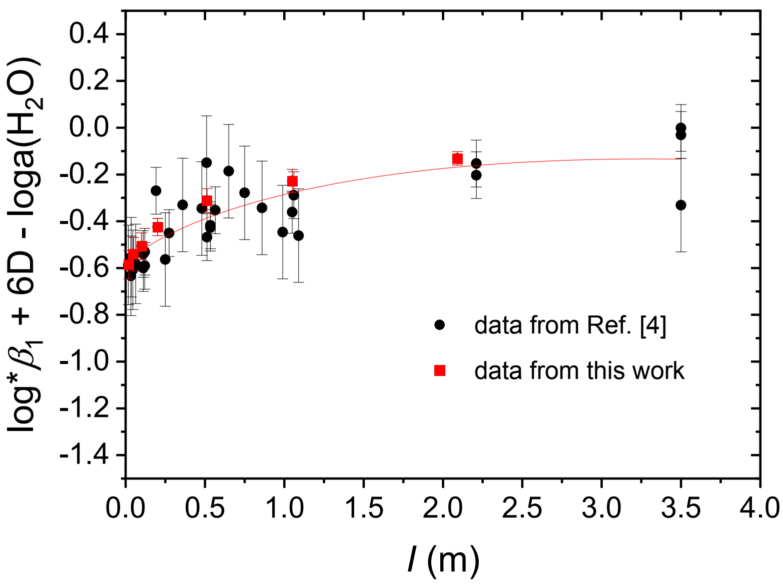

Fig. S5. Ionic strength dependence of $\log^*\beta_1$ for the first hydrolysis of U^{4+} in perchlorate media. All available stability constants at 25 °C in the literature and those in Table 3 in the main text are combined, which are listed in Table S6. The solid line is the non-linear regression fitting result for the whole data by using the two-parameter SIT model ($\Delta\varepsilon = \Delta\varepsilon_1 + \Delta\varepsilon_2 \log I$, see Eq. (S4)) as described in Ref. [4]. As a result two terms of ion-interaction coefficients ($\Delta\varepsilon_1$ and $\Delta\varepsilon_2$) and $\log^*\beta_1^\circ$ are derived and summarized in the table below.

$$Y = \log^*\beta_1 + 6D(I_m) - \log a_{H_2O} = \log^*\beta_1^{\mathrm{o}} - I_m(\Delta\varepsilon_1 + \Delta\varepsilon_2 \log I_m) \qquad \text{(S4)}$$

$\Delta\varepsilon_1$ (kg mol^{-1})	$\Delta\varepsilon_2$ (kg mol^{-1})	$\log^*\beta_1^\circ$
-(0.30 ± 0.05)	0.32 ± 0.08	-(0.59 ± 0.08)

* Note that non-weighted regression analysis and Levenberg Marquardt iteration algorithm were used.

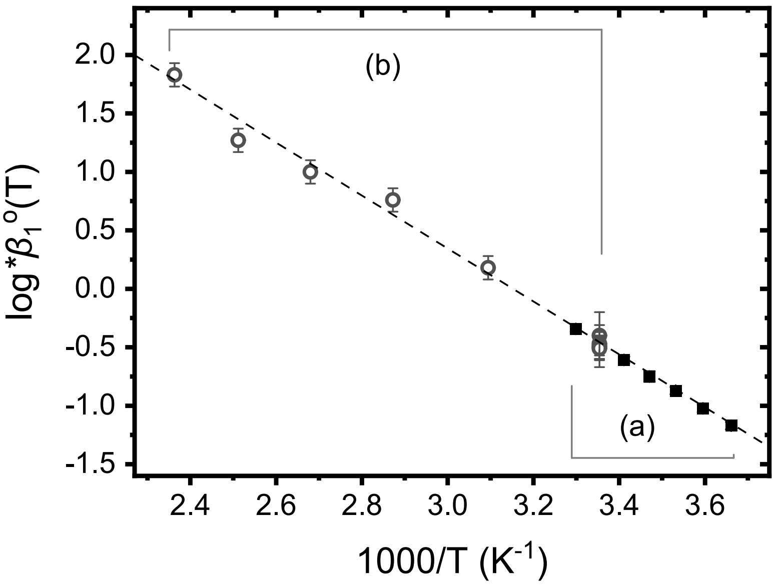

Fig. S6. Van't Hoff plot using the combined $\log^{*}\beta_1^{\circ}(T)$ data from (a) this work (Table 3 in the main text) and (b) Ref. [4] (Table 9.6, 25 – 150 °C). The dotted line indicates a weighted linear fit result. The new standard state constants, $\Delta_r H_m^{\circ}$ and $\Delta_r S_m^{\circ}$, are determined to 43.3 ± 2.2 $kJmol^{-1}$ and 136 ± 7 $JK^{-1}mol^{-1}$, respectively. The constants calculated for the data only from (b) are also shown in Table S5.

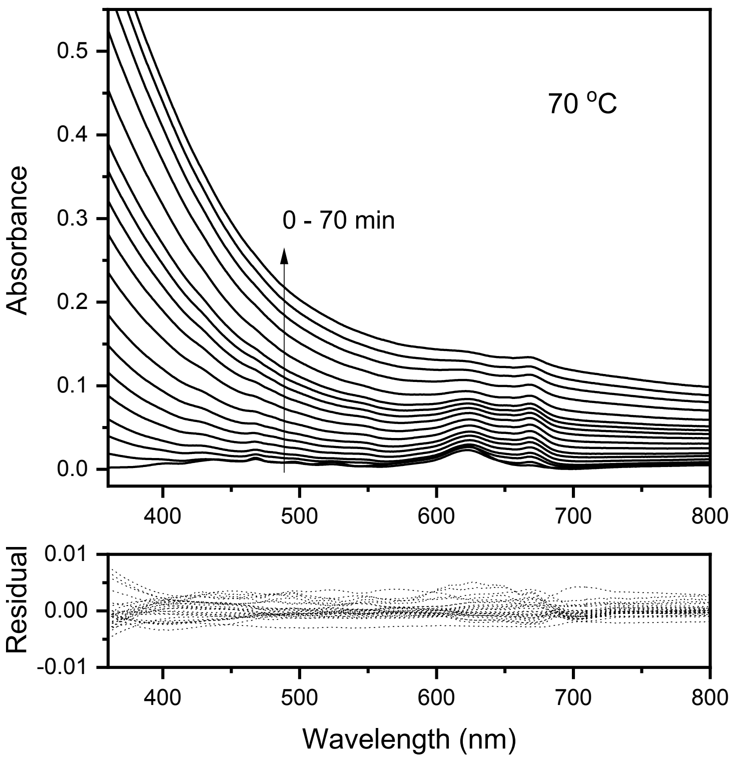

Fig. S7. Residual plot (bottom) obtained after the kinetic reaction modeling analysis of the spectrophotometric data set of Fig. 6(a) in the main text.

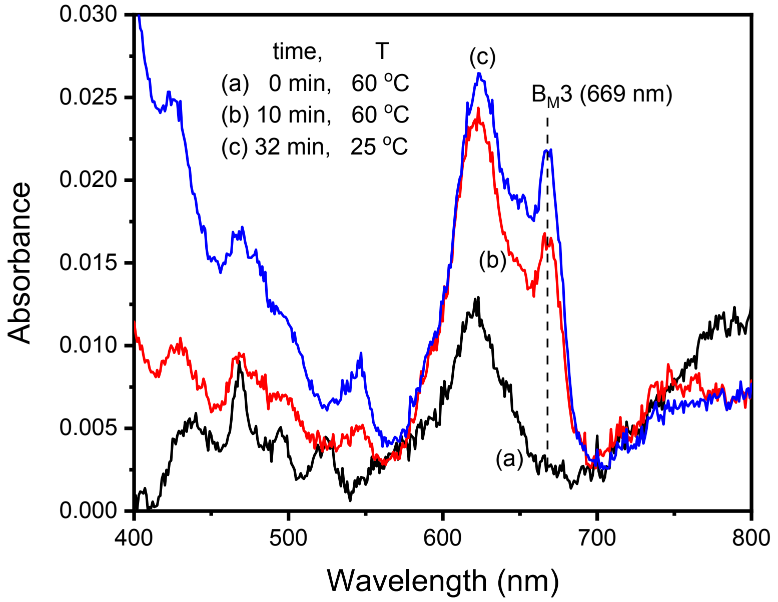

Fig. S8. Kinetic spectral data demonstrating the irreversible evolution of the intermediate (M) at 60 °C (OPL = 10 mm; [U(IV)] = 1.0 mM; pH 2.2; I = 0.1 m). The reaction temperature lowered to 25 °C after 10 min when the B_M3 band of M was evident. In the (c) spectrum the peak of the B_M3 did not disappear, instead, it increased during the period of temperature change from 60 to 25 °C. This strongly supports that the first step (k_1) of the proposed U(IV)-NP formation scheme is kinetically irreversible.

[References]

S1. R. H. Betts, *Can. J. Chem.*, 1955, **33**, 1775-1779.

S2. K.A. Kraus, F. Nelson, *J. Am. Chem. Soc.*, 1955, **77**, 3721-3722.

S3. N. M. Nikolaeva, Izv.Sib.Otd. Akad. Nauk SSSR, *Ser. Khim. Nauk*, 1978, **4**, 91-95.

S4. K.A. Kraus, F. Nelson, *J. Am. Chem. Soc.*, 1950, **72**, 3901-3906.

S5. S. Hietanen, *Acta Chem. Scand.*, 1956, **10**, 1531−1546.

S6. J.C. Sullivan, and J.C. Hindman, *J. Phys. Chem.*, 1959, **63**, 1332−1333.

S7. I. Grenthe, G. Bidoglio, N. Omenetto, *Inorg. Chem.*, 1989, **28**, 71−74.

S8. C. Ekberg, P. Brown, J. Comarmond, and Y. Albinsson, *Mater. Res. Soc. Symp. Proc.*, 2001, **663**, 1091−1099.

S9. C. Manfredi, V. Caruso, E. Vasca, S. Vero, E. Ventimiglia, G. Palladino, and D. Ferri, *J. Solution Chem.*, 2006, **35**, 927−937.
